# Economic Evidence on Biliary Tract Cancer: A Systematic Review

**DOI:** 10.3390/cancers18132057

**Published:** 2026-06-25

**Authors:** João Rocha-Gomes, Ana Sofia Teixeira, Marina Ruiz-Romeo, José Manuel Oliveira, Patrícia Ramos

**Affiliations:** 1 RISE-Health, Department of Community Medicine, Health Information and Decision (MEDCIDS), Faculty of Medicine, University of Porto, Rua Doutor Plácido da Costa, 4200-450 Porto, Portugal; 2 Ada Health GmbH, 10179 Berlin, Germany; 3 Department of Informatics Engineering, Faculty of Engineering, University of Porto, 4200-465 Porto, Portugal; st.sofiateixeira@gmail.com; 4 Department of Clinical Psychology and Psychobiology, Universitat de Barcelona, 08035 Barcelona, Spain; mrromeo@iconcologia.net; 5 ICOnnecta’t Digital Health Program, Catalan Institute of Oncology, Hospitalet de Llobregat, 08908 Barcelona, Spain; 6 Psychooncology and Digital Health Group, Bellvitge Biomedical Research Institute (IDIBELL), L’Hospitalet de Llobregat, 08908 Barcelona, Spain; 7 Institute for Systems and Computer Engineering, Technology and Science, 4200-465 Porto, Portugal; jmo@fep.up.pt (J.M.O.); patricia.ramos@inesctec.pt (P.R.); 8 Faculty of Economics, University of Porto, 4200-464 Porto, Portugal; 9 CEOS.PP, ISCAP, Polytechnic of Porto, 4465-004 São Mamede de Infesta, Portugal

**Keywords:** biliary tract cancer, immunotherapy, targeted therapy, screening, cost-effectiveness, health economics, cholangiocarcinoma, gallbladder cancer, systematic review

## Abstract

**Background**: Biliary tract cancers (BTCs), encompassing cholangiocarcinoma and gallbladder carcinoma, are aggressive malignancies with poor prognosis and increasing incidence in selected regions worldwide. Advances in imaging, biomarker profiling, immunotherapy, and targeted therapies have improved treatment options but have also increased the economic pressure on health systems. Understanding the economic evidence on BTC is therefore important for resource allocation and health technology assessment. **Methods**: We systematically searched PubMed/MEDLINE, Embase, Scopus, and Web of Science for peer-reviewed economic studies of BTC published from January 2010 to March 2025. Eligible studies included cost-effectiveness, cost–utility, cost–benefit, cost-of-illness, and resource-use analyses. The review followed PRISMA reporting principles. Reporting completeness was assessed using CHEERS 2022, and methodological credibility was appraised using the Drummond framework. **Results**: Twenty studies were included: 13 cost-effectiveness or cost–utility analyses and seven cost-of-illness or resource-use studies. Conventional chemotherapy strategies, including gemcitabine plus cisplatin in some settings and other cytotoxic combinations in selected jurisdictions, generally produced more favorable economic results than newer systemic therapies, although findings varied by country, threshold, comparator, and price assumptions. First-line immunotherapy combinations and biomarker-directed targeted therapies frequently produced ICERs above jurisdiction-specific willingness-to-pay thresholds at current prices, often requiring substantial price reductions to approach cost-effectiveness. Real-world studies showed high resource use and costs, particularly with hospitalizations and later treatment lines. Evidence on screening and prevention was limited, with one study suggesting that ultrasound surveillance may be cost-effective in a liver fluke-endemic region of Thailand. **Discussion**: The available economic evidence suggests that affordability and jurisdiction-specific value assessment are central to BTC policy decisions. Current prices for several immunotherapy and targeted agents limit cost-effectiveness in published models, while evidence on prevention, early detection, and care-pathway interventions remains sparse and context-specific.

## Simple Summary

Biliary tract cancers (BTCs) are uncommon but aggressive malignancies that are often diagnosed at an advanced stage and are associated with poor survival. New systemic therapies, including immunotherapy and biomarker-directed targeted therapies, have expanded treatment options but have also increased costs. We systematically reviewed published economic evidence on BTC, including cost-effectiveness, cost–utility, cost-of-illness, and resource-use studies. Twenty studies were included. Overall, conventional chemotherapy strategies appeared more economically favorable in several evaluated settings, although results varied by jurisdiction, model assumptions, and willingness-to-pay thresholds. In contrast, most immunotherapy combinations and targeted therapies exceeded local cost-effectiveness thresholds at current prices in the included analyses. Real-world studies showed that BTC care is resource-intensive, especially because of hospitalizations and later lines of therapy. Evidence on prevention and screening was limited, with the strongest identified example coming from ultrasound surveillance in a liver fluke-endemic region of Thailand. These findings support cautious, jurisdiction-specific health technology assessment, transparent price negotiation or managed access arrangements for high-cost therapies, and further economic evaluation of prevention, early detection, and care-pathway interventions.

## 1. Introduction

Biliary tract cancers (BTCs) are a group of malignancies arising from the epithelium of the bile ducts or gallbladder, including intrahepatic cholangiocarcinoma (iCCA), perihilar (Klatskin) and distal extrahepatic cholangiocarcinoma, and gallbladder carcinoma [1]. Collectively, BTCs are relatively rare but highly lethal. They account for approximately 3% of all gastrointestinal cancers and about 15% of primary liver malignancies [1]. The global incidence varies widely, reflecting distinct regional risk factors and genetic predispositions. Globally, an estimated 217,000 new BTC cases and 172,000 deaths occurred in 2021 [2]. Although age-standardized incidence is low in most countries (<2–3 per 100,000), some regions have much higher burdens. For example, gallbladder cancer is notably prevalent in certain areas of South Asia (northern India and Pakistan), East Asia, and Latin America (e.g., Chile and Bolivia) and is often linked to high rates of gallstone disease and chronic infections [3]. Cholangiocarcinoma has an extremely high incidence in northeast Thailand, exceeding 80 per 100,000 in some districts, due to endemic liver fluke infestation (*Opisthorchis viverrini*) [4]. Other parts of Southeast Asia and China, where liver flukes (*O. viverrini* and *Clonorchis sinensis*) are common, also have elevated cholangiocarcinoma rates [5]. In Western countries, BTCs are uncommon, but the incidence of iCCA has been increasing over recent decades [2]. For instance, iCCA mortality in the United States and Europe increased annually by approximately 3–4% in the early 21st century, possibly related to hepatitis C, nonalcoholic fatty liver disease, and other lifestyle factors [6]. Overall, BTCs rank among the top 20 causes of cancer mortality globally, and global death rates have only marginally improved despite medical advances [7]. The five-year survival remains poor (<10% in most subtypes) because of late presentation and aggressive tumor biology [8]. This epidemiological profile highlights the urgent need for better therapies and for strategies that can manage the disease burden cost-effectively worldwide.

Multiple risk factors contribute to BTC development, differing somewhat according to the anatomical subtype. For intrahepatic and extrahepatic cholangiocarcinoma, known risk factors include chronic biliary inflammation and cholestasis, such as primary sclerosing cholangitis, which confers a markedly elevated risk, hepatolithiasis, bile duct cysts, and parasitic infections or liver flukes [9]. Hepatitis B and C viral infections and cirrhosis are also associated with iCCA in some populations [10]. Gallbladder carcinoma is strongly linked to gallstone disease and chronic cholecystitis. Populations with a high prevalence of gallstones, often related to obesity or certain dietary patterns, have a higher incidence of gallbladder cancer [11]. Other contributors include high body mass index (BMI), diabetes, smoking, and possibly genetic predispositions in certain groups [12,13]. Importantly, many BTC cases remain sporadic, with no identifiable risk factor. Combined with the lack of early symptoms, this means that most patients are diagnosed at advanced, incurable stages [14]. Less than 20% of BTC patients present with resectable disease amenable to potentially curative surgery [15]. Even after curative-intent resection, recurrence rates are high and long-term survival is limited, with five-year survival commonly in the 20–40% range for resected cholangiocarcinoma [16]. These features highlight not only the clinical challenge of BTC but also the potential value of preventive efforts and early detection in high-incidence regions.

In recent years, notable advances have been made in the diagnosis and treatment of BTCs. Imaging modalities such as high-resolution magnetic resonance imaging/magnetic resonance cholangiopancreatography (MRI/MRCP) and endoscopic retrograde cholangiopancreatography (ERCP) with cholangioscopy have improved tumor detection and characterization in selected settings [17]. Novel biomarkers, including circulating tumor DNA (ctDNA) and specific genomic alterations, are under investigation for earlier diagnosis and improved prognostication [18]. Perhaps the most important progress has been in systemic therapy for advanced BTC [19]. Until 2010, there was no established chemotherapy standard [20]. The pivotal ABC-02 trial established cisplatin plus gemcitabine (GemCis) as the first-line standard of care, demonstrating a significant survival benefit over gemcitabine alone [21,22]. In the adjuvant setting after surgery, the BILCAP trial supported capecitabine as the standard adjuvant therapy for resected BTC [23]. Beyond cytotoxic chemotherapy, the treatment landscape has evolved to include targeted therapies and immunotherapy. Comprehensive genomic profiling has revealed actionable mutations in a subset of patients, including FGFR2 fusions and IDH1 mutations in intrahepatic cholangiocarcinoma [24]. These discoveries led to the development of targeted inhibitors such as pemigatinib, futibatinib, infigratinib, and ivosidenib [25,26,27]. Immune checkpoint inhibitors have also entered the field: durvalumab plus GemCis improved overall survival in TOPAZ-1, and pembrolizumab plus GemCis improved overall survival in KEYNOTE-966, albeit with relatively modest absolute survival gains [28,29]. Other approaches, such as combination chemotherapy, locoregional therapies, and liver transplantation in highly selected perihilar disease, remain clinically relevant but economically underexplored [15,30,31,32,33,34].

While these therapeutic advances are encouraging, they also bring substantial cost consequences. Novel targeted drugs and immunotherapies are expensive, often costing tens of thousands of dollars per patient [35]. BTC’s relative rarity and orphan-disease characteristics can contribute to high per-unit prices [36]. At the same time, the incremental survival gains associated with several of these agents are often measured in months rather than years, which raises legitimate concerns about cost-effectiveness [37]. BTC therefore represents an instructive case at the intersection of oncology, health economics, and policy. In many health systems, demonstrating value for money is increasingly required for adoption or reimbursement of new oncology interventions. Health technology assessment (HTA) bodies such as NICE and CADTH have already reviewed BTC therapies, including pemigatinib, and have highlighted the importance of price, uncertainty, and conditional reimbursement mechanisms in determining access [38,39]. More broadly, policymakers must decide how limited resources should be allocated between expensive treatments that benefit a biomarker-defined minority of patients and upstream measures, such as prevention, surveillance, or earlier diagnosis in high-incidence settings, that may alter the care pathway at lower cost.

In this context, it is important to systematically review the available economic evidence on BTC, including cost-effectiveness of treatments, cost-of-illness or burden studies, and economic evaluations of screening or pathway-adjacent strategies. The objectives of this review were to identify the main economic findings across BTC-related interventions and care pathways, assess how consistently economic results were reported and interpreted, and highlight important evidence gaps relevant to future research and policy. By synthesizing this literature, we aim to inform clinicians, researchers, payers, and policymakers about which strategies appear to offer better value under current evidence, where uncertainty remains substantial, and which areas of BTC care still lack robust economic evaluation.

## 2. Materials and Methods

### 2.1. Search Strategy and Selection Criteria

This systematic review was conducted and reported in accordance with PRISMA 2020 principles for study identification, screening, and reporting [40]. The review protocol was not prospectively registered. We searched PubMed/MEDLINE, Embase, Web of Science Core Collection, and Scopus for peer-reviewed studies published from 1 January 2010 to 31 March 2025. The start date of 2010 was selected because it broadly corresponds to the modern systemic-treatment era in advanced BTC, following the establishment of gemcitabine plus cisplatin as a reference first-line regimen, and because earlier economic evidence was sparse and less applicable to current clinical and policy decision-making.

The database search combined two concept blocks: (1) BTC-related terms, including biliary tract cancer, cholangiocarcinoma, gallbladder cancer, bile duct cancer, and related neoplasm terms, and (2) economic terms, including cost-effectiveness, cost–utility, cost–benefit, cost-of-illness, economic evaluation, healthcare costs, economic burden, QALY, quality-adjusted life-year, and quality of life. The full database-specific search strategies, including field tags, limits, and syntax adaptations, are provided in Supplementary Material S1. Screening, surveillance, prevention, and early-detection studies were eligible when they reported relevant economic outcomes and were retrieved through these economic search terms or through manual reference-list and targeted source checking; these concepts were not used as a separate database-search block.

No language filter was applied at the database-search stage. At eligibility assessment, however, inclusion was limited to studies available in English and with sufficient information to extract economic outcomes. Reference lists of relevant studies and selected health technology assessment sources, including NICE and CADTH documents, were also checked manually to identify additional studies not captured through the database search.

Records were deduplicated before screening. Two reviewers independently screened titles and abstracts, followed by full-text assessment of potentially eligible reports. Disagreements were resolved through discussion and consensus. Formal inter-rater agreement statistics were not prospectively recorded. Studies were eligible if they: (1) focused on BTC, including cholangiocarcinoma or gallbladder carcinoma; (2) reported economic outcomes, including cost-effectiveness, cost–utility, cost–benefit, cost-of-illness, resource use, ICERs, cost per QALY, cost per life-year gained, total costs, or incremental costs; and (3) were original peer-reviewed research, including model-based economic evaluations, trial-based economic analyses, or observational cost/resource-use studies. We excluded editorials, commentaries, narrative reviews without original economic analysis, conference abstracts without sufficient full data, studies without BTC-specific results, and studies that mentioned costs only tangentially without formal economic analysis.

### 2.2. Data Extraction and Critical Appraisal

Data were extracted using a standardized extraction form. Extracted variables included author, year, country or region, study design, population, sample size, intervention and comparator, study perspective, model structure where applicable, time horizon, discount rate, cost categories, price year and currency where reported, clinical-effectiveness inputs, utility inputs, main economic outcomes, sensitivity analyses, and authors’ conclusions. For cost-effectiveness and cost–utility studies, we extracted ICERs, QALYs, life-years, total and incremental costs, willingness-to-pay thresholds, and the probability of cost-effectiveness where available. For cost-of-illness and resource-use studies, we extracted mean or median costs, cost categories, healthcare utilization, hospitalizations, treatment-line costs, productivity outcomes where reported, and principal cost drivers.

Data extraction was performed using the standardized form and checked by a second reviewer against the source publications for consistency. Disagreements or uncertainties were resolved through discussion. Study authors were not routinely contacted for clarification, and the review relied on data available in the published reports and associated Supplementary Materials.

Reporting completeness of included economic evaluations was assessed using the CHEERS 2022 checklist [41], while methodological credibility was appraised using the Drummond criteria for economic evaluations [42]. CHEERS was used as a reporting framework rather than as a risk-of-bias scoring tool. The Drummond framework was used to assess whether the economic question, comparator, perspective, cost and outcome measurement, valuation, discounting, uncertainty analysis, and interpretation were appropriately addressed. Appraisal was summarized at the study level in Supplementary Material S3. No study was excluded solely on the basis of appraisal findings; instead, appraisal informed the narrative weighting of evidence, with greater interpretive weight given to studies using transparent methods, appropriate comparators, robust clinical inputs, and adequate uncertainty analysis.

### 2.3. Synthesis and Cost Presentation

Given the heterogeneity of study designs, interventions, comparators, jurisdictions, perspectives, and outcomes, meta-analysis was not appropriate. We therefore conducted a narrative synthesis. Included studies were grouped into the following categories: (1) first-line systemic therapies for advanced BTC; (2) second-line and biomarker-driven therapies; (3) screening, prevention, and pathway-adjacent diagnostic strategies; and (4) cost-of-illness and real-world resource-use studies.

We compared findings within each category, emphasizing the decision context, comparator, jurisdiction, willingness-to-pay threshold, and price assumptions used in each study. Cost-effectiveness conclusions were interpreted within the original jurisdictional context and were not treated as universally transferable across health systems.

Monetary values were extracted and reported primarily in the currencies and price contexts used by the original studies. Where original studies reported converted values, these were retained. Where additional review-level conversions were used for orientation, they were documented separately in Supplementary Material S4, including original currency, price year where available, exchange-rate or inflation assumptions, and whether the conversion was author-reported or calculated by the review authors. Converted values were used only to aid interpretation and were not treated as a fully standardized basis for ranking interventions across countries.

## 3. Results

### 3.1. Study Selection and Characteristics

**Figure 1:**
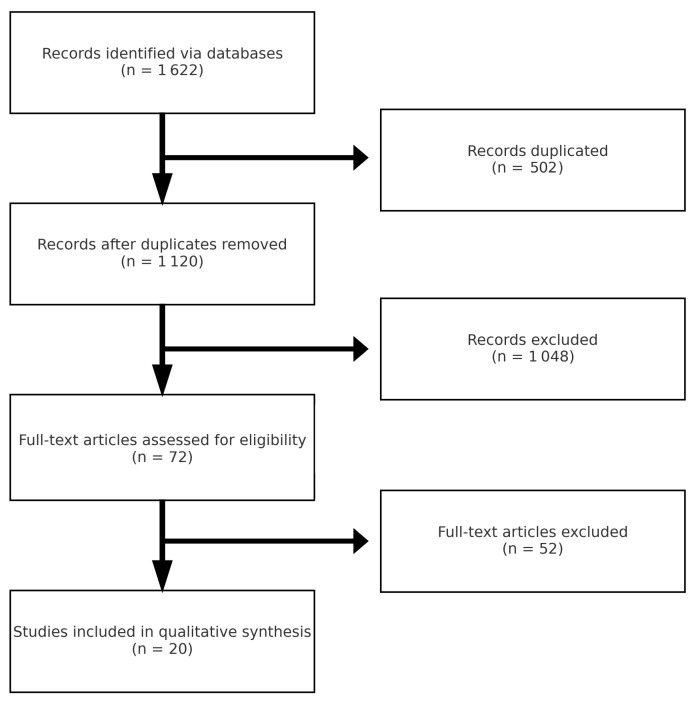
PRISMA 2020 flow diagram of study selection. The diagram separates database records from manual or other-source records and shows the number of records at each stage of identification, deduplication, screening, eligibility assessment, and inclusion. A total of 20 studies were included in this systematic review. Full-text exclusion details are provided in Supplementary Material S3. Figure created by the authors.

The eligible literature was dominated by model-based cost-effectiveness and cost–utility evaluations of systemic therapies for advanced or metastatic BTC. A smaller number of studies examined screening, diagnostic, or pathway-adjacent strategies, and seven studies provided cost-of-illness or real-world resource-use evidence. The study selection process is summarized in Figure 1. The search retrieved 1622 records, including 1612 from databases and 10 from manual searching. After removing 502 duplicates, 1120 titles and abstracts were screened; 1048 records were excluded because they were unrelated to BTC or did not contain an economic analysis. We assessed 72 full-text articles and excluded 52 for the following reasons: not BTC-specific (*n* = 12), no original economic data (*n* = 8), duplicate or superseded analyses (*n* = 10), conference abstracts without sufficient full data (*n* = 8), non-English full text (*n* = 5), or other methodological limitations that precluded meaningful synthesis (*n* = 9). Twenty studies met the inclusion criteria and were synthesized narratively.

The evidence base was geographically diverse: seven studies originated in East Asia (China, Taiwan, or Japan), one in Southeast Asia (Thailand), five in the United States, three in Europe (Spain, Sweden, and the Netherlands), and one in Canada, and three were multi-regional analyses covering both the United States and China. Most cost-effectiveness studies adopted a healthcare-payer or healthcare-system perspective, while only a minority explicitly incorporated broader societal costs such as productivity loss. Eighteen studies focused on cholangiocarcinoma or mixed BTC cohorts, whereas two studies focused on incidental gallbladder cancer detected after cholecystectomy for presumed benign disease.

Broadly, the included studies suggest that conventional chemotherapy regimens and some pathway-focused strategies can be economically favorable in selected jurisdictions, whereas most current-price immunotherapies and targeted agents are not cost-effective under the assumptions used in the jurisdictions studied. The burden-of-illness studies consistently show that BTC care is resource-intensive and that costs typically rise with disease progression, repeated hospitalization, and later lines of therapy. These broad patterns, however, should always be interpreted within the context of each study’s perspective, comparator, threshold, and price assumptions (Table 1).

### 3.2. First-Line Therapies in Advanced BTC

**Figure 2:**
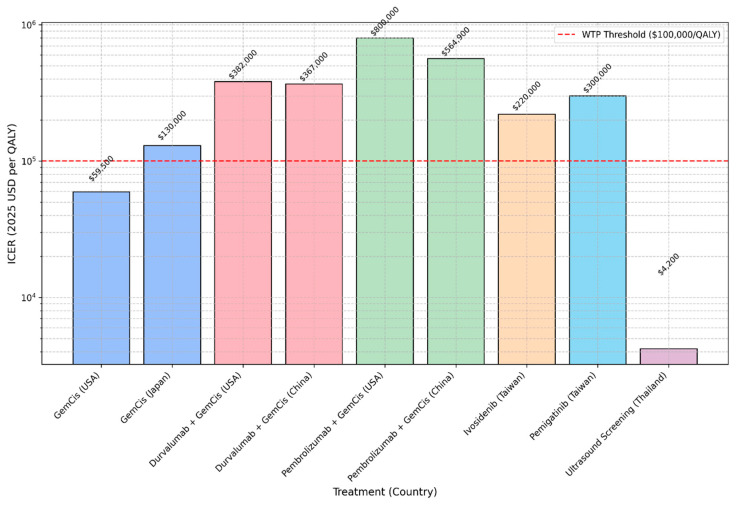
Reported cost-effectiveness results for selected biliary tract cancer interventions. Bars show ICERs reported by included studies or 2024 USD-equivalent orientation values documented in Supplementary Material S4. Bar colors distinguish intervention categories: conventional chemotherapy regimens, immunotherapy combinations, targeted therapies, and screening/surveillance strategies. No universal willingness-to-pay threshold is shown because thresholds differ across jurisdictions. Values should be interpreted within the original jurisdiction, payer perspective, price year, comparator, and willingness-to-pay threshold used in each study. The figure is intended as a visual overview rather than a fully standardized cross-country ranking. Figure created by the authors.

Economic evidence for first-line systemic therapy was mixed and clearly context-dependent. In the United States, Roth and Carlson found that gemcitabine plus cisplatin (GemCis) versus gemcitabine alone produced an ICER of approximately US $59,480/QALY, which fell within commonly cited U.S. willingness-to-pay ranges [56]. By contrast, Tsukiyama et al. reported that the same comparison was not cost-effective in Japan at the thresholds applied in that study, illustrating how the perceived value of an established regimen can change across settings [57]. Chen et al. further showed in China that capecitabine plus oxaliplatin (XELOX) could dominate gemcitabine plus oxaliplatin (GEMOX) by generating slightly more QALYs at lower total cost, suggesting that some conventional chemotherapy choices may be economically attractive when prices, comparators, and local practice patterns differ [44]. A systematic review and network meta-analysis of first-line chemotherapy regimens also highlighted variation in efficacy and toxicity across regimens, reinforcing the importance of comparator selection when interpreting economic findings [49] (Figure 2).

The newer first-line immunotherapy combinations were consistently associated with substantially higher ICERs in the included economic evaluations. Across studies from China, the United States, and Japan, pembrolizumab plus GemCis and durvalumab plus GemCis were generally not cost-effective at current prices under the thresholds applied in those analyses [37,49,50,53,60,61]. These findings should be interpreted alongside the clinical evidence from TOPAZ-1 and KEYNOTE-966, which showed statistically significant but modest absolute survival improvements when durvalumab or pembrolizumab was added to GemCis [28,29]. In the economic models, the cost of the immune-checkpoint inhibitor component was repeatedly identified as a major driver of unfavorable cost-effectiveness results.

An important nuance is that not all multi-drug first-line strategies were uniformly unfavorable. Kashiwa and Maeda, using a Japanese payer perspective, found that the GCS triplet, consisting of gemcitabine, cisplatin, and S-1, could be cost-effective, whereas the durvalumab- and pembrolizumab-based combinations were not cost-effective under the assumptions tested [49]. Taken together, the first-line evidence suggests that context matters substantially: some conventional or regionally adapted chemotherapy regimens may represent good value, whereas currently priced immune-checkpoint combinations generally do not meet jurisdiction-specific cost-effectiveness thresholds in the available analyses.

### 3.3. Second-Line and Biomarker-Driven Therapy

The second-line economic literature was relatively sparse and concentrated on biomarker-selected therapies rather than on cytotoxic chemotherapy. For IDH1-mutant advanced intrahepatic cholangiocarcinoma, Chen et al. evaluated ivosidenib in Taiwan and found that it generated modest incremental QALY gains compared with mFOLFOX or 5-FU/LV, with ICERs above the country’s willingness-to-pay threshold [45]. Their analysis suggested that substantial price reductions would be required before ivosidenib would become economically attractive under Taiwanese payer assumptions. These economic findings are consistent with the clinical evidence from ClarIDHy, which confirmed a benefit for ivosidenib in IDH1-mutant cholangiocarcinoma but with limited absolute gains in a previously treated population [27,62].

A similar pattern was observed for pemigatinib in FGFR2-fusion cholangiocarcinoma. Chueh et al. found that pemigatinib was not cost-effective at the hypothesized price compared with mFOLFOX or 5-FU/LV, although substantial price reductions improved the probability of cost-effectiveness [47]. These findings are also consistent with HTA decisions in Canada and the United Kingdom, where reimbursement discussions for pemigatinib reflected both clinical need and uncertainty regarding cost-effectiveness at list price [38,39]. The underlying clinical rationale for pemigatinib is supported by the development of FGFR-targeted treatment in molecularly selected cholangiocarcinoma, but the economic evidence indicates that price and uncertainty remain central to value assessment [24,26].

By contrast, we did not identify a dedicated peer-reviewed cost-effectiveness analysis of FOLFOX versus active symptom control in BTC, despite FOLFOX becoming a clinically relevant second-line cytotoxic option after ABC-06 and subsequent synthesis supporting its use in selected patients previously treated with cisplatin/gemcitabine [63,64]. This absence is noteworthy because it leaves a gap between clinical standard-of-care evidence and formal economic evaluation. Overall, the second-line evidence supports a cautious interpretation: targeted agents may offer clinically relevant options for molecularly selected patients, but at current prices, they have generally not been shown to be cost-effective in the jurisdictions studied.

### 3.4. Adjuvant, Curative-Intent, and Pathway-Adjacent Evidence

For adjuvant and curative-intent settings, direct BTC-specific economic evidence was limited. The BILCAP trial established adjuvant capecitabine as an important treatment option after resection [23], yet we did not identify a dedicated formal economic evaluation of adjuvant capecitabine in BTC. Likewise, although surgery remains the principal curative treatment for resectable disease [16] and liver transplantation is an option for highly selected patients with perihilar cholangiocarcinoma [34], we found no BTC-specific cost-effectiveness analyses of surgery or transplantation as treatment classes. This distinction is important: the available evidence does not support a generalized statement that curative-intent BTC care is cost-effective. Rather, it highlights an evidence gap in the economic evaluation of resection, transplantation, and perioperative care pathways.

Two included studies addressed pathway-adjacent economic evidence related to incidental gallbladder cancer detection after cholecystectomy for presumed benign disease. Olthof et al. and Lundgren et al. evaluated whether routine histopathological examination of all gallbladder specimens was economically justified [52,54]. Although these studies do not evaluate the treatment of established BTC directly, they are relevant to BTC-related diagnostic pathways. Both analyses suggested that selective histopathology strategies could reduce unnecessary spending with minimal loss of clinically important detection in low-risk benign-disease populations. Because these studies address a different decision point from advanced BTC therapy, we treated them separately in the synthesis rather than allowing them to drive treatment-level conclusions.

Beyond these examples, we found little direct economic evidence on other pathways or supportive interventions, including radiotherapy, photodynamic therapy, radiofrequency ablation, or perioperative strategies. The existing clinical literature describes some of these approaches in selected palliative or locoregional contexts [32,33,65,66], but we did not identify BTC-specific cost-effectiveness analyses sufficient for synthesis. This reinforces that the BTC economic literature remains concentrated in advanced systemic treatment, with comparatively limited evaluation of earlier-stage, supportive, locoregional, or pathway-level interventions.

Only one included study directly evaluated a BTC-specific screening or early-detection strategy. In a high-incidence area of Thailand, Laopachee et al. found that ultrasound surveillance among high-risk adults generated modest QALY gains at an ICER below the local willingness-to-pay threshold, while also increasing the proportion of cancers detected at Stage I [51]. Within that specific endemic setting, ultrasound surveillance therefore appeared economically favorable.

At the same time, the prevention and early-detection evidence base was otherwise limited. We did not identify eligible BTC-specific economic evaluations of mass liver fluke eradication, hepatitis B vaccination, hepatitis C treatment, or other primary-prevention strategies that could be directly synthesized within this review. These interventions remain biologically and public-health relevant to BTC risk, but stronger claims about their BTC-specific economic value would go beyond the included evidence. Programmatic infrastructure such as CASCAP and broader liver fluke control efforts illustrate the public-health relevance of this agenda in endemic settings [67,68], but the appropriate conclusion is that prevention-oriented economic evidence is promising but currently narrow and context-specific.

### 3.5. Real-World Resource Use and Costs

Six studies described real-world costs, healthcare utilization, or productivity burden, and together they portray BTC care as resource-intensive [43,46,48,55,58,59]. In the United States, Wadhwa et al. used inpatient data to show that annual admissions for cholangiocarcinoma increased over time and that inpatient care imposed a substantial hospital burden [58]. Chamberlain et al., using claims data, reported substantial per-patient-per-month costs among patients with advanced cholangiocarcinoma, reflecting the high cost of medical services, treatment, and disease management [43]. Parasuraman et al. reported monthly healthcare costs of approximately US $10,000–US $11,000 among working-age patients and also demonstrated measurable productivity losses, showing that BTC has both direct and indirect economic consequences [55]. Wang et al. further showed that healthcare utilization and costs increased across treatment lines in advanced BTC, with later lines associated with higher per-patient-per-month expenditure [59].

Outside the United States, Choi et al. characterized the hospital burden of BTC across 28 hospitals in Ontario, Canada, while Darbà and Marsà reported hospital incidence and direct medical costs for intrahepatic cholangiocarcinoma in Spain [46,48]. These studies suggest that BTC creates a material healthcare burden across different health-system contexts, although absolute cost estimates vary substantially because of differences in payer structures, unit prices, hospital financing, and data sources.

Quality-of-life implications are also relevant to BTC economics. Advanced BTC is associated with significant symptom burden, including jaundice, pruritus, pain, fatigue, and treatment-related toxicity [69]. Economic evaluations therefore depend heavily on utility estimates and assumptions about progression-free versus progressed-disease quality of life. However, BTC-specific utility evidence remains limited, and some models rely on utility values from broader oncology sources rather than BTC-specific preference studies [70]. This represents an additional source of uncertainty in current model-based evaluations.

## 4. Discussion

This review synthesizes an emerging but still methodologically uneven body of economic evidence on BTC. Several broad patterns are visible. First, the literature is strongly concentrated on advanced systemic therapy, especially first-line chemotherapy, first-line immunotherapy combinations, and a small number of targeted therapies for biomarker-selected disease. Second, real-world cost studies consistently suggest that BTC imposes a substantial economic burden, driven by hospital utilization, repeated treatment, disease progression, and poor survival. Third, important evidence gaps remain in curative-intent, adjuvant, supportive, locoregional, and prevention-oriented care.

A central finding is that most current-price immunotherapies and targeted agents were not cost-effective in the jurisdictions studied. This does not mean that these agents lack clinical value. Rather, it means that under the model assumptions, thresholds, price levels, and comparators used in the available analyses, the incremental benefit was generally not large enough to justify the incremental cost [37,45,47,49,50,53,60,61]. That distinction matters. Cost-effectiveness in BTC is highly context-specific, and results from one jurisdiction should not be generalized uncritically to another. Nonetheless, the direction of findings was consistent: list-price immunotherapy and precision-oncology regimens often struggled to meet willingness-to-pay thresholds in the evaluated settings.

By contrast, several conventional regimens and pathway-focused interventions appeared more economically favorable. GemCis, XELOX in China, and the GCS triplet in Japan performed better economically than most higher-cost novel regimens in the included studies [44,50,56,57]. However, even here, the evidence should not be oversimplified. This review does not support a broad claim that all standard BTC care is cost-effective as a class. Instead, it supports the narrower conclusion that some established regimens appear to represent better value than currently priced novel therapies in specific evaluated settings. The same caution applies to surgery and curative-intent care, where the economic evidence base remains limited despite the clinical importance of these pathways [16,23,34].

The burden-of-illness and resource-use studies add an important real-world perspective to the cost-effectiveness literature. Model-based analyses can appear abstract when expressed as ICERs, but claims and hospital data show what BTC means in practice: costly admissions, expensive systemic therapy, lost productivity, and escalating expenditure as disease progresses [43,46,48,55,58,59]. Even though BTC is less common than several other malignancies, its per-patient burden can be considerable. This is particularly relevant for health systems attempting to reconcile orphan-cancer innovation with finite budgets.

Regional variation in value assessment also emerged clearly. Thresholds, drug prices, financing arrangements, clinical practice patterns, and health-system capacity differ substantially across the United States, Europe, and Asia. A regimen that appears acceptable in one setting may be unaffordable or poor value in another. This is especially important for BTC because many evaluated interventions are expensive and involve relatively modest absolute survival gains. As a result, the external validity of any single ICER is inherently limited. Cross-country comparison is still useful, but mainly for identifying patterns and drivers rather than for establishing a universal hierarchy of value.

The prevention and early-detection findings require particular restraint. The Thai ultrasound-surveillance study is important because it shows that, under conditions of very high local risk and an organized surveillance context, early detection can be economically attractive [51]. However, it remains the only directly included BTC-specific prevention-oriented economic evaluation. We therefore interpret it as evidence from one high-risk endemic setting rather than as a basis for broad generalization. Broader policy discussions around liver fluke control, endemic-area screening infrastructure such as CASCAP, vaccination, or other prevention strategies remain relevant [67,68], but stronger BTC-specific economic evidence is needed before firm conclusions can be drawn within this review framework.

The review also raises equity questions. BTC burden is not distributed evenly. In some settings, populations facing the highest risk may also have limited access to specialist diagnosis, molecular testing, surgery, or expensive systemic therapy. This creates a tension between precision-oncology advances and population-level affordability. From a policy standpoint, the present evidence supports careful use of jurisdiction-specific HTAs, value-based pricing, managed access agreements, and further evidence development for high-cost agents. It also highlights the need to evaluate earlier and potentially more equitable interventions in high-risk communities. The epidemiology of actionable molecular alterations is unevenly characterized across BTC populations, which has implications for both clinical access and economic evaluation [71].

### 4.1. Limitations

This review has several limitations. First, it was not prospectively registered. Second, although screening and full-text review were conducted independently by two reviewers, we did not record a formal inter-rater agreement statistic, and the review process would have been strengthened by prospective protocol registration and fuller workflow documentation. Third, data extraction and appraisal were checked by a second reviewer, but not all stages were conducted as fully duplicate independent processes. Fourth, final inclusion was restricted to English-language full-text publications with extractable economic data, which may have introduced language bias and may have excluded regional evidence from high-incidence settings.

Fifth, the search strategy focused on BTC terms combined with economic terms. Screening, surveillance, prevention, and early-detection studies were eligible if they reported economic outcomes and were captured through these terms or through manual checking, but the database strategy did not include a separate prevention or screening search block. Relevant prevention-focused economic studies may therefore have been missed if they were not indexed or described using economic terminology. Sixth, the included evidence was conceptually heterogeneous, spanning first-line therapy, biomarker-selected second-line therapy, screening, incidental gallbladder pathology strategies, and burden-of-illness studies. This heterogeneity limited the possibility of formal quantitative synthesis.

Seventh, many included studies were model-based economic evaluations that depended on extrapolation beyond observed trial follow-up, structural assumptions, drug prices, and non-BTC-specific utility or cost inputs. These choices can materially affect ICERs. Eighth, although we sought to improve cross-study readability by documenting selected monetary conversions, direct comparison across jurisdictions remains difficult because willingness-to-pay thresholds, price years, model structures, purchasing power, and reimbursement contexts differ substantially. Converted monetary values should therefore be interpreted only as orientation aids rather than as fully standardized cross-country estimates. Ninth, the prevention and early-detection evidence base was very limited, so conclusions in that area must remain cautious. Finally, although we supplemented database searching with manual review of references and selected HTA sources, we did not conduct a fully systematic gray-literature search, so unpublished or non-indexed economic evidence may have been missed.

### 4.2. Future Directions

Future BTC economic research should improve both breadth and methodological depth. First, clinically important areas still lack dedicated economic evaluation, including adjuvant therapy, resection pathways, transplantation in highly selected disease, supportive and palliative interventions, locoregional therapies, and treatment sequencing, particularly as first-line and later-line systemic options continue to evolve clinically [72] and despite economic evaluations being feasible in other selected oncologic transplant indications [73]. Second, future trials and real-world studies of new BTC regimens should embed quality-of-life and resource-use collection prospectively so that economic analyses do not depend so heavily on post hoc modeling or assumptions borrowed from other cancers [74]. Third, molecularly stratified BTC research should be linked more explicitly to real-world biomarker prevalence and access patterns so that economic evaluations better reflect the populations in whom targeted strategies are actually implementable [71].

Fourth, more prevention- and early-detection research is needed, particularly in endemic or otherwise high-risk settings where the underlying disease burden may make earlier intervention economically attractive. The Thai ultrasound-surveillance study provides a useful example, but comparable economic evaluations are needed in other risk groups and health-system contexts [51]. Fifth, more BTC-specific utility studies would improve model credibility, as would greater transparency regarding price year, exchange-rate source, inflation adjustment, and structural uncertainty. Finally, future systematic reviews in this field would benefit from a prospectively registered protocol, fully reproducible search reporting, and study-level supplementary appraisal tables so that readers can better judge both reporting completeness and methodological credibility.

## 5. Conclusions

This systematic review shows that the BTC economic literature remains relatively small, treatment-focused, and highly context-dependent. Across the included studies, conventional chemotherapy regimens and selected pathway-oriented strategies tended to appear more economically favorable than most current-price immunotherapy or biomarker-selected targeted therapies, although these conclusions varied by jurisdiction and should not be generalized beyond the assumptions of the evaluated studies.

The review also shows that BTC care is costly in real-world practice and that expenditure tends to rise with disease progression and later lines of therapy. At the same time, the evidence base remains thin in several areas of major clinical importance, including curative-intent care, adjuvant therapy, supportive interventions, locoregional treatment, and prevention or early detection. Future work should therefore aim not only to evaluate new technologies but also to fill the upstream and pathway-level evidence gaps that matter for sustainable BTC care.

Overall, improving BTC outcomes sustainably will require both effective therapies and robust evidence that these interventions can be delivered equitably and affordably. Jurisdiction-specific HTAs, transparent modeling, price negotiation, managed access arrangements, and stronger real-world evidence will be central to aligning innovation with value in BTC care, particularly as health systems face increasing pressure to assess the value of high-cost anticancer drugs [75].

## Figures and Tables

**Table 1:** Included studies on economic evaluations and cost-of-illness studies in biliary tract cancer.

Count	Author (Year)	Country/Setting	Study Design	Intervention(s) vs. Comparator(s)	Population/Perspective	Key Findings
1	Chamberlain CX et al. (2021) [43]	USA (Optum Clinformatics^®^ Data Mart claims; Optum, Eden Prairie, MN, USA; 2007-2019)	Cost-of-illness (retrospective cohort)	N/A (descriptive cost-of-illness—no formal comparator)	*n* = 1298 adults (mean age 69.1 years) with advanced cholangiocarcinoma (73.8% intrahepatic, 21.2% extrahepatic); identified via Medicare Advantage or commercial insurance databases; US payer perspective	Mean cost per patient-month was US $7743 (medical services US $6685; drug costs US $1058); median OS was 5.3 months; nearly 40% did not receive second-line therapy, while among those who did: 40.3% received fluoropyrimidine-based regimens, 30.7% gemcitabine-based regimens, and 29.3% capecitabine—highlighting high costs and unmet treatment needs.
2	Chen et al. (2022) [44]	China (payer perspective)	Partitioned-survival CEA (Markov model)	XELOX (capecitabine + oxaliplatin) vs. GEMOX (gemcitabine + oxaliplatin)	Adults with advanced biliary tract cancer, modeled on a phase III non-inferiority RCT, including *n* = 222 patients (108 XELOX; 114 GEMOX); Chinese National Health Insurance perspective	XELOX yielded higher QALYs (0.66 vs. 0.54) and lower total cost (US $12,275.51) than GEMOX—producing a dominant strategy. Sensitivity analyses showed >92% probability of cost-effectiveness at 1–3× GDP thresholds.
3	Chen et al. (2024) [45]	Taiwan (NHI payer)	Partitioned survival CEA (3-state model)	Ivosidenib vs. mFOLFOX vs. 5 FU/LV	Hypothetical cohort based on the ClarIDHy trial (*n* = 187) of previously treated adults with IDH1-mutant advanced intrahepatic cholangiocarcinoma; Taiwanese National Health Insurance perspective	ICER vs. mFOLFOX: NT $6,268,528/QALY; vs. 5-FU/LV: NT $5,670,555/QALY—both exceed 3× GDP WTP. PSA showed price cuts (~50–60%) needed (NT $4161–5201/500 mg) for cost-effectiveness.
4	Choi et al. (2024) [46]	Canada (Ontario, 28 GEMINI hospitals)	Retrospective cohort; administrative data audit	Descriptive (Subgroup comparison: iCCA vs. eCCA vs. GBC)	*n* = 4596 BTC-related hospitalizations among 3102 unique patients (2720 iCCA; 1269 eCCA; 607 GBC); hospital perspective	iCCA admissions increased by 23% (from 385 to 420, *p* = 0.005). Median cost per BTC hospitalization in 2021 was CAD 8507 (IQR CAD 5416 to CAD 16,152). The 2024 USD-equivalent orientation value documented in Supplementary Material S4 is approximately US $7053 per admission. Inflation-adjusted BTC hospital spending increased by 18%; length of stay was broadly stable.
5	Chueh et al. (2023) [47]	Taiwan (NHIA payer; 3-state partitioned-survival model)	Partitioned-survival CEA + PSA	Pemigatinib vs. 5-FU/LV & mFOLFOX in sequence	Hypothetical cohort based on the FIGHT-202 phase II trial (*n* = 107) of advanced FGFR2-fusion intrahepatic cholangiocarcinoma patients; Taiwanese National Health Insurance perspective	Pemigatinib was not cost-effective at the hypothesized price of NT $17,820/13.5 mg. The ICER versus mFOLFOX and 5-FU was NT $3,411,098/QALY (approximately US $111,300/QALY as a 2024 USD-equivalent orientation value in Supplementary Material S4). PSA showed approximately 53% (mFOLFOX) and 57% (5-FU) probability of cost-effectiveness. A price reduction of at least approximately 50% improved cost-effectiveness results.
6	Darbà & Marsà (2021) [48]	Spain (National hospital discharge database, 2000–2018)	Retrospective cost-of-illness (hospital-level)	N/A (observational; descriptive)	*n* = 23,315 hospital admissions for intrahepatic cholangiocarcinoma, median age 73 years (IQR 17), 55.9% male; hospital perspective	Incidence increased to 6.9/10,000 in 2018. Mean cost plateaued at approximately €9417/patient after 2009. In-hospital mortality remained substantial at 31.5% in 2018 despite higher incidence. Median length of stay was 10 days and decreased significantly over time.
7	Jiang et al. (2025) [49]	China & USA (Markov model)	Cost–utility CEA (3-state)	Pembrolizumab + Gem/Cis vs. Gem/Cis alone	Hypothetical cohort matching the KEYNOTE-966 trial (*n* = 1069) of advanced BTC patients (ECOG 0–1); US and Chinese payer perspectives	ICERs were US $810,184/QALY (US) and US $360,933/QALY (China), both exceeding the applied willingness-to-pay thresholds. The combination was not cost-effective in either setting; pembrolizumab cost was the most influential parameter in sensitivity analyses.
8	Kashiwa & Maeda (2024) [50]	Japan (10 year partitioned survival model)	Cost effectiveness CEA (Markov)	Gemcitabine + cisplatin + S 1 (GCS) vs. Gem/Cis; Durvalumab combo (DGC); Pembrolizumab combo (PGC)	Hypothetical cohort based on trial populations: KHBO1401-MITSUBA (*n* = 377), TOPAZ-1 (*n* = 685), and KEYNOTE-966 (*n* = 1069) representing first-line advanced BTC patients from Japanese healthcare payer perspective	GCS yielded 1.63 QALYs and an ICER of approximately ¥3.78 million/QALY, suggesting cost-effectiveness in the Japanese payer setting. DGC (¥86.06 million/QALY) and PGC (¥28.98 million/QALY) were not cost-effective under the assumptions tested.
9	Laopachee et al. (2023) [51]	Thailand (screening cohort)	Cost-effective analysis (decision-tree CEA)	Ultrasound surveillance vs. no screening	High-risk adults aged 30–60 years in CCA-endemic northern Thailand: *n* = 4225 in surveillance cohort and *n* = 121 in non-surveillance; societal perspective	QALY gain was 0.117 QALYs. The ICER was approximately THB 152,985/QALY (approximately US $4407/QALY as a 2024 USD-equivalent orientation value in Supplementary Material S4). Early detection substantially increased Stage I diagnosis (34% vs. 8%). The ICER was close to or below the Thai willingness-to-pay threshold used by the authors.
10	Lundgren et al. (2020) [52]	Sweden	CEA (decision tree)	Strategy comparisons: Routine histology vs. macroscopic selective vs. current selective vs. none	Real-world data of *n* = 81,349 cholecystectomies informing a hypothetical cohort of 10,000 patients undergoing cholecystectomy for benign gallbladder disease; Swedish healthcare payer perspective	A selective histopathology strategy was cost-saving in the modeled pathway; routine histology added minimal life-year gains at high incremental cost. The reported ICER was €76,508/LY versus routine pathology in the relevant comparison.
11	Luo X et al. (2024) [53]	China & USA (Markov model, based on KEYNOTE 966)	Cost–utility CEA	Pembrolizumab + Gem/Cis vs. Gem/Cis alone	Hypothetical cohorts matching the KEYNOTE-966 trial (*n* = 1069) for Chinese and US advanced BTC patients; payer perspectives	China: ΔQALY +0.14 and ΔCost +US $77,115, giving an ICER of US $556,689/QALY, above the applied WTP threshold. US: ΔQALY +0.14 and ΔCost +US $160,425, giving an ICER of US $1,109,462.92/QALY, above the applied threshold; INHB = −0.25 QALYs.
12	Olthof et al. (2018) [54]	Netherlands (single center, 2011–2017)	Retrospective cost analysis	Routine pathology ± automatic follow-up vs. macroscopic selective	*n* = 2763 adult patients undergoing cholecystectomy for benign gallstone disease; societal perspective	Routine pathology generated approximately €160,000/year in potentially avoidable expenditure in the single-center setting. A macroscopic selective policy could save approximately €25,000/year with an estimated risk of missed cancers below 0.1%.
13	Parasuraman (2023) [55]	USA (Optum, Eden Prairie, MN, USA—Health claims data, 2017–2021)	Retrospective cost-of-illness and productivity loss analysis	Subgroup comparison (iCCA vs. eCCA vs. GBC)	*n* = 1065 adults with cholangiocarcinoma (624 iCCA, 380 eCCA, 61 both); working-age, full-time employees with ≥1 month post-index; payer & societal perspectives	Mean monthly healthcare cost was approximately US $10,300 to US $11,200. iCCA patients incurred higher costs and greater work loss than eCCA or GBC. Indirect costs from missed work were approximately US $622 to US $690/month, highlighting both direct and indirect economic burden.
14	Roth & Carlson (2012) [56]	USA (societal)	CEA (Markov)	Gemcitabine + cisplatin vs. gemcitabine	Hypothetical cohort of *n* = 410 patients mirroring ABC-02 trial demographics (locally advanced/metastatic BTC; ECOG 0–2); US societal perspective	Incremental QALYs were +0.19 (0.751 vs. 0.561). The ICER was US $59,480/QALY, below commonly cited US WTP thresholds. Results were sensitive to survival, utilities, and progression costs; PSA showed GemCis was cost-effective when WTP exceeded approximately US $60,000/QALY.
15	Tsukiyama et al. (2017) [57]	Japan (BT 22 trial-based)	CEA (Markov)	Gemcitabine + cisplatin vs. gemcitabine	Hypothetical cohort modeled from the BT-22 trial (*n* = 83) of Japanese patients with advanced BTC (ECOG 0–1); Japanese healthcare payer perspective	ICER was approximately ¥13.7 million/QALY, above the Japanese WTP threshold used in the study (approximately ¥5–6 million/QALY). Deterministic sensitivity analysis was robust; probability of cost-effectiveness was below 33% at a ¥6 million/QALY threshold.
16	Wadhwa et al. (2017) [58]	USA (National Inpatient Sample, 1997–2012)	Retrospective cohort analysis	N/A (observational; descriptive)	All U.S. hospital discharges with principal diagnosis of cholangiocarcinoma: 10,357 admissions in 1997, rising to 11,970 in 2012; U.S. hospital charges perspective	Admissions increased by ~16% (10,357 → 11,970; *p* < 0.001). Mean hospital charges rose 113%: from US $36,460 → $77,753 (inflation-adjusted, *p* < 0.001). Length of stay decreased from 9.5 → 7.9 days (*p* < 0.001). In-hospital mortality dropped from 9.3% → 6.4% (*p* < 0.001).
17	Wang et al. (2024) [59]	USA (integrated EHR claims, HIRD^®^ Carelon Research, Wilmington, DE, USA—linked to CCQP)	Retrospective cohort analysis	N/A (observational; descriptive—Treatment line progression 1L vs. 2L vs. 3L)	*n* = 413 patients, median age 61, 55% female; stage III–IV advanced BTC; U.S. payer perspective	Median OS was 11.5 months. PPPM all-cause costs increased across treatment lines: 1L US $19,589, 2L US $22,617, and 3L US $33,534 (SDs 22,603, 19,302, and 40,588). Up to approximately 70% had at least one inpatient admission per line.
18	Ye et al. (2023) [37]	USA & China	CEA (Markov)	Durvalumab + GemCis vs. GemCis alone	Hypothetical cohort representing patients from the TOPAZ-1 trial (*n* = 685); payer perspective in US and Chinese healthcare systems	ICER was US $381,864/QALY from the US payer perspective and US $367,609/QALY from the Chinese payer perspective. Both ICERs exceeded country-specific WTP thresholds. Sensitivity analyses showed that results were strongly influenced by durvalumab price.
19	Zhao et al. (2023) [60]	China	CEA (3-state partitioned-survival)	Durvalumab + Gem/Cis vs. Gem/Cis	Hypothetical cohort of first-line advanced BTC patients, with population modeled from the TOPAZ-1 trial (*n* = 685); Chinese payer perspective	Incremental QALYs were +0.12 and incremental costs were approximately US $18,555. ICER with charity assistance was approximately US $159,645/QALY; without assistance, approximately US $696,571/QALY. Both exceeded China’s WTP threshold (approximately US $37,663/QALY); a large price reduction was required to meet the threshold.
20	Zheng et al. (2023) [61]	China	Cost–utility analysis (Markov-like)	Pembrolizumab + Gem/Cis vs. Gem/Cis alone	Hypothetical cohort based on KEYNOTE 966 trial: *n* = 1069 first-line advanced BTC patients (ECOG 0–1), Chinese payer perspective	ΔQALYs = +0.184 and ΔCosts ≈ US $103,941. ICER ≈ US $564,895/QALY, well above China’s WTP threshold (approximately US $37,304/QALY). Pembrolizumab cost, progressed-disease utility, and subsequent treatment costs were the most influential parameters; no scenario fell below the threshold.

Monetary values are reported in the currency and price context used by the original studies unless otherwise specified. Review-level conversions, when used for orientation, are documented in Supplementary Material S4 and should not be interpreted as fully standardized cross-country comparisons. BTC = biliary tract cancer; CCA = cholangiocarcinoma; iCCA = intrahepatic cholangiocarcinoma; eCCA = extrahepatic cholangiocarcinoma; GBC = gallbladder cancer; 1L = first-line therapy; 2L = second-line therapy; 3L = third-line therapy; Gem = gemcitabine; Cis = cisplatin; 5-FU = 5-fluorouracil; LV = leucovorin; mFOLFOX = modified FOLFOX (5-FU + oxaliplatin ± leucovorin); QALY = quality-adjusted life-year; ICER = incremental cost-effectiveness ratio; WTP = willingness-to-pay threshold; NHI = National Health Insurance; NHIA = National Health Insurance Administration; CEA = cost-effectiveness analysis; PSA = probabilistic sensitivity analysis; PPPM = per-patient per-month; OS = overall survival; EHR = electronic health records; INHB = incremental net health benefit. All monetary values are in 2025 US $ unless stated otherwise; models adopt payer or societal perspectives, as indicated.

## Data Availability

No primary patient-level data were generated or analyzed in this study. Extracted study-level data and full-text exclusion details are available in Supplementary Material S3, and cost-currency/conversion notes are available in Supplementary Material S4.

## Supplementary Materials

The following supporting information can be downloaded at https://www.mdpi.com/article/10.3390/cancers18132057/s1. References [40,41,42,76,77,78,79] are cited in the Supplementary Materials.

## References

[1] Marcano-Bonilla L., Mohamed E.A., Mounajjed T., Roberts L.R. (2016). Biliary tract cancers: Epidemiology, molecular pathogenesis and genetic risk associations. Chin. Clin. Oncol..

[2] Xie D., Liu F., Zhou D., Zhu Q., Xiao F., Zhang K. (2025). Global burden and cross-country inequalities in gallbladder and biliary tract cancer (1990–2021) with projections to 2050: Insights from the Global Burden of Disease Study 2021. Front. Med..

[3] Hundal R., Shaffer E.A. (2014). Gallbladder cancer: Epidemiology and outcome. Clin. Epidemiol..

[4] Sahat O., Bilheem S., Lim A., Kamsa-Ard S., Thinkhamrop Suwannatrai A., Uadrang S., Leklob A., Chansaard W., Sriket N., Santong C. (2025). Updated cholangiocarcinoma incidence trends and projections in Thailand by region based on data from four population-based cancer registries. Lancet Reg. Health Southeast Asia.

[5] Sithithaworn P., Yongvanit P., Duenngai K., Kiatsopit N., Pairojkul C. (2014). Roles of liver fluke infection as risk factor for cholangiocarcinoma. J. Hepato-Biliary-Pancreat. Sci..

[6] Bertuccio P., Malvezzi M., Carioli G., Hashim D., Boffetta P., El-Serag H.B., La Vecchia C., Negri E. (2019). Global trends in mortality from intrahepatic and extrahepatic cholangiocarcinoma. J. Hepatol..

[7] Baria K., De Toni E.N., Yu B., Jiang Z., Kabadi S.M., Malvezzi M. (2022). Worldwide incidence and mortality of biliary tract cancer. Gastro Hep Adv..

[8] Goetze T.O., Roderburg C., Friedrich F.W., Trojan J. (2024). New perspectives in biliary tract cancers. ESMO Gastrointest. Oncol..

[9] Khan S.A., Tavolari S., Brandi G. (2019). Cholangiocarcinoma: Epidemiology and risk factors. Liver Int..

[10] Veracruz N., Gish R.G., Cheung R., Chitnis A.S., Wong R.J. (2022). Global incidence and mortality of hepatitis B and hepatitis C acute infections, cirrhosis and hepatocellular carcinoma from 2010 to 2019. J. Viral Hepat..

[11] Stinton L.M., Shaffer E.A. (2012). Epidemiology of gallbladder disease: Cholelithiasis and cancer. Gut Liver.

[12] Hu Z., Wang X., Zhang X., Sun W., Mao J. (2024). An analysis of the global burden of gallbladder and biliary tract cancer attributable to high BMI in 204 countries and territories: 1990–2021. Front. Nutr..

[13] Park J.-H., Hong J.Y., Park Y.S., Kang G., Han K., Park J.O. (2021). Association of prediabetes, diabetes, and diabetes duration with biliary tract cancer risk: A nationwide cohort study. Metabolism.

[14] Ghidini M., Pizzo C., Botticelli A., Hahne J.C., Passalacqua R., Tomasello G., Petrelli F. (2018). Biliary tract cancer: Current challenges and future prospects. Cancer Manag. Res..

[15] Oneda E., Abu Hilal M., Zaniboni A. (2020). Biliary tract cancer: Current medical treatment strategies. Cancers.

[16] Cillo U., Fondevila C., Donadon M., Gringeri E., Mocchegiani F., Schlitt H.J., Ijzermans J.N.M., Vivarelli M., Zieniewicz K., Olde Damink S.W.M. (2019). Surgery for cholangiocarcinoma. Liver Int..

[17] Park H.S., Lee J.M., Choi J.Y., Lee M.W., Kim H.J., Han J.K. (2008). Preoperative evaluation of bile duct cancer: MRI combined with MR cholangiopancreatography versus MDCT with direct cholangiography. AJR Am. J. Roentgenol..

[18] Rizzo A., Ricci A.D., Tavolari S., Brandi G. (2020). Circulating tumor DNA in biliary tract cancer: Current evidence and future perspectives. Cancer Genom. Proteom..

[19] Xie C., McGrath N.A., Monge Bonilla C., Fu J. (2020). Systemic treatment options for advanced biliary tract carcinoma. J. Gastroenterol..

[20] Filippi R., Lombardi P., Quara V., Fenocchio E., Aimar G., Milanesio M., Leone F., Aglietta M. (2019). Pharmacotherapeutic options for biliary tract cancer: Current standard of care and new perspectives. Expert Opin. Pharmacother..

[21] Valle J., Wasan H., Palmer D.H., Cunningham D., Anthoney A., Maraveyas A., Madhusudan S., Iveson T., Hughes S., Pereira S.P. (2010). Cisplatin plus gemcitabine versus gemcitabine for biliary tract cancer. N. Engl. J. Med..

[22] Valle J., Wasan H., Palmer D.H., Cunningham D., Anthoney A., Maraveyas A., Madhusudan S., Iveson T., Hughes S., Pereira S.P. (2009). Gemcitabine with or without cisplatin in patients with advanced or metastatic biliary tract cancer (ABC): Results of a multicenter, randomized phase III trial (the UK ABC-02 trial). J. Clin. Oncol..

[23] Primrose J.N., Fox R.P., Palmer D.H., Malik H.Z., Prasad R., Mirza D., Anthony A., Corrie P., Falk S., Finch-Jones M. (2019). Capecitabine compared with observation in resected biliary tract cancer (BILCAP): A randomised, controlled, multicentre, phase 3 study. Lancet Oncol..

[24] Goyal L., Kongpetch S., Crolley V.E., Bridgewater J. (2021). Targeting FGFR inhibition in cholangiocarcinoma. Cancer Treat. Rev..

[25] Deiana C., Ricci C., Vahabi M., Ali M., Brandi G., Giovannetti E. (2024). Advances in target drugs and immunotherapy for biliary tract cancer. Expert Rev. Gastroenterol. Hepatol..

[26] Patel T.H., Marcus L., Horiba M.N., Donoghue M., Chatterjee S., Mishra-Kalyani P.S., Schuck R.N., Li Y., Zhang X., Fourie Zirkelbach J. (2023). FDA approval summary: Pemigatinib for previously treated, unresectable locally advanced or metastatic cholangiocarcinoma with FGFR2 fusion or other rearrangement. Clin. Cancer Res..

[27] Abou-Alfa G.K., Macarulla T., Javle M.M., Kelley R.K., Lubner S.J., Adeva J., Cleary J.M., Catenacci D.V., Borad M.J., Bridgewater J. (2020). Ivosidenib in IDH1-mutant, chemotherapy-refractory cholangiocarcinoma (ClarIDHy): A multicentre, randomised, double-blind, placebo-controlled, phase 3 study. Lancet Oncol..

[28] Oh D.-Y., He A.R., Bouattour M., Okusaka T., Qin S., Chen L.-T., Kitano M., Lee C.-K., Kim J.W., Chen M.-H. (2024). Durvalumab or placebo plus gemcitabine and cisplatin in participants with advanced biliary tract cancer (TOPAZ-1): Updated overall survival from a randomised phase 3 study. Lancet Gastroenterol. Hepatol..

[29] Kelley R.K., Ueno M., Yoo C., Finn R.S., Furuse J., Ren Z., Yau T., Klumpen H.J., Chan S.L., Ozaka M. (2023). Pembrolizumab in combination with gemcitabine and cisplatin compared with gemcitabine and cisplatin alone for patients with advanced biliary tract cancer (KEYNOTE-966): A randomised, double-blind, placebo-controlled, phase 3 trial. Lancet.

[30] Greenhalgh J., Mahon J., Bryning S., Chaplin M., Beale S., Boland A., Dundar Y., McEntee J., Basu B. (2023). Durvalumab with Gemcitabine and Cisplatin for Treating Unresectable or Advanced Biliary Tract Cancer [ID4031].

[31] Banales J.M., Marin J.J.G., Lamarca A., Rodrigues P.M., Khan S.A., Roberts L.R., Cardinale V., Braconi C., Calvisi D.F., Perugorria M.J. (2020). Cholangiocarcinoma 2020: The next horizon in mechanisms and management. Nat. Rev. Gastroenterol. Hepatol..

[32] Talreja J.P., Kahaleh M. (2010). Photodynamic therapy for cholangiocarcinoma. Gut Liver.

[33] Brass V., Kuhlmann J.B., Blum H.E. (2014). Current state of nonsurgical therapies for cholangiocarcinoma. Hepatic Oncol..

[34] Borakati A., Froghi F., Bhogal R.H., Mavroeidis V.K. (2023). Liver transplantation in the management of cholangiocarcinoma: Evolution and contemporary advances. World J. Gastroenterol..

[35] Schaft N., Dorrie J., Schuler G., Schuler-Thurner B., Sallam H., Klein S., Eisenberg G., Frankenburg S., Lotem M., Khatib A. (2023). The future of affordable cancer immunotherapy. Front. Immunol..

[36] Danzon P.M. (2018). Affordability challenges to value-based pricing: Mass diseases, orphan diseases, and cures. Value Health.

[37] Ye Z.-M., Xu Z., Li H., Li Q. (2023). Cost-effectiveness analysis of durvalumab plus chemotherapy as first-line treatment for biliary tract cancer. Front. Public Health.

[38] Reimbursement Team (2022). Pemigatinib (Pemazyre). Can. J. Health Technol..

[39] NICE (2021). Pemigatinib for Treating Relapsed or Refractory Advanced Cholangiocarcinoma with FGFR2 Fusion or Rearrangement.

[40] Page M.J., McKenzie J.E., Bossuyt P.M., Boutron I., Hoffmann T.C., Mulrow C.D., Shamseer L., Tetzlaff J.M., Akl E.A., Brennan S.E. (2021). The PRISMA 2020 statement: An updated guideline for reporting systematic reviews. BMJ.

[41] Husereau D., Drummond M., Augustovski F., de Bekker-Grob E., Briggs A.H., Carswell C., Caulley L., Chaiyakunapruk N., Greenberg D., Loder E. (2022). Consolidated Health Economic Evaluation Reporting Standards 2022 (CHEERS 2022) Statement: Updated Reporting Guidance for Health Economic Evaluations. BMJ.

[42] Drummond M.F., Sculpher M.J., Claxton K., Stoddart G.L., Torrance G.W. (2015). Methods for the Economic Evaluation of Health Care Programmes.

[43] Chamberlain C.X., Faust E., Goldschmidt D., Webster N., Boscoe A.N., Macaulay D., Peters M.L.B. (2021). Burden of illness for patients with cholangiocarcinoma in the United States: A retrospective claims analysis. J. Gastrointest. Oncol..

[44] Chen R., Zhang Y., Lin K., Huang D., You M., Lai Y., Wang J., Hu Y., Li N. (2022). Cost-effectiveness analysis of capecitabine plus oxaliplatin versus gemcitabine plus oxaliplatin as first-line therapy for advanced biliary tract cancers. Front. Pharmacol..

[45] Chen K.A., Huang W.M., Chen E.Y.T., Ho P.K., Chueh C.H., Wen Y.W., Chen M.H., Chiang N.J., Tsai Y.W. (2024). Cost-effectiveness of ivosidenib versus chemotherapy for previously treated IDH1-mutant advanced intrahepatic cholangiocarcinoma in Taiwan. BMC Cancer.

[46] Choi W.J., Roberts S., Verma A., Razak F., O’Kane G.M., Gallinger S., Hirschfield G., Hansen B., Sapisochin G. (2024). Characterizing the burden of biliary tract cancers across 28 hospitals in Ontario, Canada. Cancer.

[47] Chueh C.H., Tsai Y.-W., Chen Z.R., Shiu M.N., Wen Y.W., Chiang N.J. (2023). Cost-effectiveness analysis of a new second-line treatment regimen for advanced intrahepatic cholangiocarcinoma: Biomarker-driven targeted therapy of pemigatinib versus 5-FU chemotherapy. PharmacoEconomics.

[48] Darbà J., Marsà A. (2021). Analysis of hospital incidence and direct medical costs of intrahepatic cholangiocarcinoma in Spain (2000–2018). Expert Rev. Pharmacoecon. Outcomes Res..

[49] Jiang C., Zhou K., Shu P. (2025). Cost-effectiveness analysis of pembrolizumab plus chemotherapy as first-line treatment for advanced biliary tract cancer: Perspectives from US and Chinese payers. BMJ Open.

[50] Kashiwa M., Maeda H. (2024). Comparative Cost-Effectiveness of Gemcitabine and Cisplatin in Combination with S-1, Durvalumab, or Pembrolizumab as First-Line Triple Treatment for Advanced Biliary Tract Cancer. J. Gastrointest. Cancer.

[51] Laopachee P., Siripongsakun S., Sangmala P., Chanree P., Hiranrat P., Srisittimongkon S. (2023). Cost-effectiveness analysis of ultrasound surveillance for cholangiocarcinoma in an endemic area of Thailand. Asian Pac. J. Cancer Prev..

[52] Lundgren L., Henriksson M., Andersson B., Sandstrom P. (2020). Cost-effectiveness of gallbladder histopathology after cholecystectomy for benign disease. BJS Open.

[53] Luo X., Cai T., Wu J., Li X., Wang X., Ma H. (2024). Cost-effectiveness of pembrolizumab plus chemotherapy vs. chemotherapy as first-line treatment for advanced biliary tract cancer in China and the US. Front. Pharmacol..

[54] Olthof P.B., Metman M.J.H., de Krijger R.R., Scheepers J.J., Roos D., Dekker J.W.T. (2018). Routine pathology and postoperative follow-up are not cost-effective in cholecystectomy for benign gallbladder disease. World J. Surg..

[55] Parasuraman S., Thiel E., Park J., Teschemaker A. (2023). Productivity loss outcomes and costs among patients with cholangiocarcinoma in the United States: An economic evaluation. J. Med. Econ..

[56] Roth J.A., Carlson J.J. (2012). Cost-effectiveness of gemcitabine + cisplatin vs. gemcitabine monotherapy in advanced biliary tract cancer. J. Gastrointest. Cancer.

[57] Tsukiyama I., Ejiri M., Yamamoto Y., Nakao H., Yoneda M., Matsuura K., Arakawa I., Saito H., Inoue T. (2017). A cost-effectiveness analysis of gemcitabine plus cisplatin versus gemcitabine alone for treatment of advanced biliary tract cancer in Japan. J. Gastrointest. Cancer.

[58] Wadhwa V., Jobanputra Y., Thota P.N., Menon K.V., Parsi M.A., Sanaka M.R. (2017). Healthcare utilization and costs associated with cholangiocarcinoma. Gastroenterol. Rep..

[59] Wang L., Singhal M., Valderrama A., Nepal B., Kamble S., Eluri M., Malhotra U., Siegel A.B., Grabner M., Swami S. (2024). Real-world treatment patterns, resource utilization and costs in biliary tract cancers in the USA. Future Oncol..

[60] Zhao Q., Xie R., Zhong W., Liu W., Chen T., Qiu X., Yang L. (2023). Cost-effectiveness analysis of adding durvalumab to chemotherapy as first-line treatment for advanced biliary tract cancer based on the TOPAZ-1 trial. Cost Eff. Resour. Alloc..

[61] Zheng Z., Fang L., Cai H. (2023). Cost-effectiveness analysis of pembrolizumab in combination with chemotherapy compared with chemotherapy alone as first-line treatment for patients with advanced biliary tract cancer in China. BMC Cancer.

[62] Zhu A.X., Macarulla T., Javle M.M., Kelley R.K., Lubner S.J., Adeva J., Cleary J.M., Catenacci D.V.T., Borad M.J., Bridgewater J.A. (2021). Final overall survival efficacy results of ivosidenib for patients with advanced cholangiocarcinoma with IDH1 mutation: The phase 3 randomized clinical ClarIDHy trial. JAMA Oncol..

[63] Lamarca A., Palmer D.H., Wasan H.S., Ross P.J., Ma Y.T., Arora A., Falk S., Gillmore R., Wadsley J., Patel K. (2021). Second-line FOLFOX chemotherapy versus active symptom control for advanced biliary tract cancer (ABC-06): A phase 3, open-label, randomised, controlled trial. Lancet Oncol..

[64] Digklia A., Arnold D., Voutsadakis I.A. (2024). Second-line FOLFOX chemotherapy for patients with advanced biliary tract cancers pretreated with cisplatin/gemcitabine: A systematic review and meta-analysis. ESMO Gastrointest. Oncol..

[65] Mohan B.P., Chandan S., Khan S.R., Kassab L.L., Ponnada S., Artifon E.L.A., Otoch J.P., McDonough S., Adler D.G. (2022). Photodynamic therapy (PDT), radiofrequency ablation (RFA) with biliary stents in palliative treatment of unresectable extrahepatic cholangiocarcinoma: A systematic review and meta-analysis. J. Clin. Gastroenterol..

[66] Aebisher D., Rogoz K., Mysliwiec A., Dynarowicz K., Wiench R., Cieslar G., Kawczyk-Krupka A., Bartusik-Aebisher D. (2024). The use of photodynamic therapy in medical practice. Front. Oncol..

[67] Khuntikeo N., Chamadol N., Yongvanit P., Loilome W., Namwat N., Sithithaworn P., Andrews R.H., Petney T.N., Promthet S., Thinkhamrop K. (2015). Cohort profile: Cholangiocarcinoma screening and care program (CASCAP). BMC Cancer.

[68] Wongba N., Thaewnongiew K., Phathee K., Laithavewat L., Duangsong R., Promthet S., Tangsawad S. (2011). Liver fluke prevention and control in the northeast of Thailand through action research. Asian Pac. J. Cancer Prev..

[69] Hunter L.A., Soares H.P. (2021). Quality of life and symptom management in advanced biliary tract cancers. Cancers.

[70] Pourrahmat M.M., Kansal A.R., Chung K.C., Hux M., Howarth A., Fazeli M.S. (2020). Health state utility values across cancer types and stages: A systematic literature review. Value Health.

[71] Kratz J.D., Klein A.B., Gray C.B., Marten A., Vilu H.-L., Knight J.F., Kumichel A., Ueno M. (2024). The epidemiology of biliary tract cancer and associated prevalence of MDM2 amplification: A targeted literature review. Target. Oncol..

[72] Jiang Y., Zeng Z., Zeng J., Liu C., Qiu J., Li Y., Tang J., Mo N., Du L., Ma J. (2021). Efficacy and safety of first-line chemotherapies for patients with advanced biliary tract carcinoma: A systematic review and network meta-analysis. Front. Oncol..

[73] Jung K., Park J., Jung J.H., Lee J.-C., Kim J., Hwang J.-H. (2022). Real-world outcomes of gemcitabine, cisplatin, and nab-paclitaxel chemotherapy regimen for advanced biliary tract cancer: A propensity score-matched analysis. Gut Liver.

[74] Bjornelv G.M.W., Dueland S., Line P.-D., Joranger P., Fretland A.A., Edwin B., Sorbye H., Aas E. (2019). Cost-effectiveness of liver transplantation in patients with colorectal metastases confined to the liver. Br. J. Surg..

[75] Stewart D.J., Bradford J.-P., Sehdev S., Ramsay T., Navani V., Rawson N.S.B., Jiang D.M., Gotfrit J., Wheatley-Price P., Liu G. (2024). New anticancer drugs: Reliably assessing “value” while addressing high prices. Curr. Oncol..

[76] World Bank World Development Indicators: Consumer Price Index (FP.CPI.TOTL). https://data.worldbank.org/indicator/FP.CPI.TOTL.

[77] Board of Governors of the Federal Reserve System Foreign Exchange Rates: G.5A Annual Release. https://www.federalreserve.gov/releases/g5a/current/.

[78] Directorate-General of Budget, Accounting and Statistics, Executive Yuan, Taiwan Consumer Price Index. https://eng.dgbas.gov.tw/.

[79] Bank of Thailand, Ministry of Commerce, Thailand Headline Consumer Price Index Annual Change. https://www.bot.or.th/.

