# Supplementary material for: Economic Evidence on Biliary Tract Cancer: A Systematic Review
Source: Cancers (Basel). 2026 Jun 25;18(13):2057. doi: 10.3390/cancers18132057 (PMC13360021; doi:10.3390/cancers18132057)
Supplement: Supplementary file 1 [file cancers-18-02057-s001.zip › Supplementary_Material_S1_Search Queries.pdf]

Supplementary Material S1 — Complete Database Search Strategies

Economic evidence on biliary tract cancer: a systematic review

Overview

This supplement provides the complete database search strategies used to identify studies on the economics of biliary tract cancer (BTC). Four bibliographic databases were searched: PubMed/MEDLINE, Embase via Embase.com, Web of Science Core Collection, and Scopus.

A single conceptual search strategy was developed and translated into the syntax of each database. The strategy combined two concept blocks:

- Block 1, population: biliary tract cancer and related terms, including cholangiocarcinoma, gallbladder cancer, bile duct cancer, and biliary tract neoplasm terms.
- Block 2, economic outcomes: cost-effectiveness, cost-utility, cost-benefit, cost-of-illness, economic evaluation, healthcare costs, economic burden, QALY, quality-adjusted life-year, and quality-of-life terms.

Block 1 was combined with Block 2 using the Boolean operator AND. Within each block, synonyms were combined using OR. Field tags, database-specific syntax, publication-year limits, and document-type or subject-area filters were adapted to each database as shown below. Controlled-vocabulary terms such as MeSH or Emtree headings were not used as separate search blocks.

Screening, surveillance, prevention, and early-detection studies were eligible when they reported relevant economic outcomes and were retrieved through the economic search block or through manual reference-list and targeted source checking. These concepts were not included as a separate database-search block.

The search strategy reported here corresponds to the search described in the Methods section of the manuscript. It is provided to support reproducibility under PRISMA 2020 item 7.

Common search limits and eligibility handling

- Publication date: 1 January 2010 to 31 March 2025.
- Search execution date: March 2025.
- Language: no language filter was applied at the database-search stage. English-language availability was applied during eligibility assessment, as described in the manuscript.

- Document type: original peer-reviewed articles were prioritized. Editorials, commentaries, narrative reviews without original economic analysis, and conference abstracts without sufficient extractable data were excluded during screening or full-text assessment.
- Manual checking: reference lists of relevant studies and selected health technology assessment sources, including NICE and CADTH documents, were checked manually to identify additional eligible studies not captured through the database search.

S1.1 PubMed / MEDLINE

Interface: PubMed (NCBI), https://pubmed.ncbi.nlm.nih.gov/ **Date of search:** March 2025 **Field tags used:** default PubMed search fields; [PDAT] for publication date.

("biliary tract cancer" OR "biliary tract neoplasm" OR cholangiocarcinoma OR "gallbladder cancer" OR "gallbladder neoplasm*" OR "bile duct cancer")
AND
("cost-effectiveness" OR "cost effectiveness" OR "cost-utility" OR "cost utility" OR "cost-benefit" OR "cost benefit" OR "cost-of-illness" OR "cost of illness" OR "economic evaluation" OR "healthcare costs" OR "health care costs" OR "economic burden" OR QALY OR "quality-adjusted life year" OR "quality of life")
AND 2010:2025[PDAT]

This is the complete PubMed/MEDLINE query underlying the summarized search description reported in the Methods section of the manuscript.

Notes on adaptation:

- PubMed was searched using free-text/default-field terms and the publication-date field tag [PDAT].
- No separate MeSH controlled-vocabulary block was applied.
- The date filter 2010:2025[PDAT] captured the target publication-year range. Records outside the final date window of 1 January 2010 to 31 March 2025, if retrieved, were excluded during screening.

S1.2 Embase (via Embase.com)

Interface: Embase.com (Elsevier), https://www.embase.com/ **Date of search:** March 2025 **Field tags used:** :ti,ab,kw for title, abstract, and keywords; [2010-2025]/py for publication year range.

('biliary tract cancer':ti,ab,kw OR 'biliary tract neoplasm':ti,ab,kw OR 'biliary tract neoplasms':ti,ab,kw OR cholangiocarcinoma:ti,ab,kw OR cholangiocarcinomas:ti,ab,kw OR 'gallbladder cancer':ti,ab,kw OR 'gallbladder neoplasm*':ti,ab,kw OR 'bile duct cancer':ti,ab,kw)
AND
('cost-effectiveness':ti,ab,kw OR 'cost effectiveness':ti,ab,kw OR 'cost-utility':ti,ab,kw OR 'cost utility':ti,ab,kw OR 'cost-benefit':ti,ab,kw OR 'cost benefit':ti,ab,kw OR 'cost-of-illness':ti,ab,kw OR 'cost of illness':ti,ab,kw OR 'economic evaluation':ti,ab,kw OR 'healthcare costs':ti,ab,kw OR 'health care costs':ti,ab,kw OR 'economic burden':ti,ab,kw OR QALY:ti,ab,kw OR 'quality-adjusted life year':ti,ab,kw OR 'quality of life':ti,ab,kw)
AND [2010-2025]/py

Notes on adaptation:

- Multi-word terms in Embase.com were enclosed in single quotation marks.
- The wildcard * was retained in 'gallbladder neoplasm*' to match both singular and plural indexed forms.
- The tag :ti,ab,kw restricts matching to title, abstract, and author keywords, which is the closest functional equivalent of the PubMed free-text search.
- [2010-2025]/py applies the publication-year filter.
- No separate Emtree controlled-vocabulary block was applied.

S1.3 Web of Science Core Collection

Interface: Web of Science, Clarivate, https://www.webofscience.com/ **Collection queried:** Web of Science Core Collection (SCI-EXPANDED, SSCI, ESCI) **Date of search:** March 2025 **Field tag used:** TS= (Topic: title, abstract, author keywords, Keywords Plus).

TS=(("biliary tract cancer" OR "biliary tract neoplasm" OR cholangiocarcinoma OR "gallbladder cancer" OR "gallbladd er neoplasm*" OR "bile duct cancer")
AND
("cost-effectiveness" OR "cost effectiveness" OR "cost-utility" OR "cost utility" OR "cost-benefit" OR "cost benefit" OR " cost-of-illness" OR "cost of illness" OR "economic evaluation" OR "healthcare costs" OR "health care costs" OR "econo mic burden" OR QALY OR "quality-adjusted life year" OR "quality of life"))

Refinements (applied via the interface):

- Timespan: 2010-01-01 to 2025-03-31.
- Document Types: Article.

Notes on adaptation:

- TS= is the standard topic-field operator in Web of Science and is the closest equivalent to PubMed's default all-fields behaviour combined with Embase's :ti,ab,kw.
- The wildcard * is supported natively and was preserved for "gallbladder neoplasm*".
- Hyphens inside phrases are treated variably across databases; both hyphenated and non-hyphenated forms, such as "cost-effectiveness" and "cost effectiveness", were retained to maintain cross-database consistency.
- Web of Science does not use MeSH or Emtree indexing; the search was therefore implemented using Topic fields.

S1.4 Scopus

Interface: Scopus (Elsevier), https://www.scopus.com/ **Date of search:** March 2025 **Field operator used:** TITLE-ABS-KEY() for title, abstract, and keywords; PUBYEAR for year filter; SUBJAREA for subject-area filter; DOCTYPE for document type.

TITLE-ABS-KEY(
("biliary tract cancer" OR "biliary tract neoplasm" OR cholangiocarcinoma OR "gallbladder cancer" OR "gallbladder neoplasm*" OR "bile duct cancer")
AND
("cost-effectiveness" OR "cost effectiveness" OR "cost-utility" OR "cost utility" OR "cost-benefit" OR "cost benefit" OR

"cost-of-illness" OR "cost of illness" OR "economic evaluation" OR "healthcare costs" OR "health care costs" OR "econo mic burden" OR QALY OR "quality-adjusted life year" OR "quality of life")
)
AND PUBYEAR > 2009 AND PUBYEAR < 2026
AND (LIMIT-TO(SUBJAREA, "MEDI") OR LIMIT-TO(SUBJAREA, "HEAL") OR LIMIT-TO(SUBJAREA, "NURS") OR LIMIT-TO(SUBJAREA, "PHAR"))
AND (LIMIT-TO(DOCTYPE, "ar"))

Notes on adaptation:

- TITLE-ABS-KEY() restricts the search to title, abstract, and keyword fields.
- Health-related subject-area codes were applied to improve specificity: MEDI for Medicine, HEAL for Health Professions, NURS for Nursing, and PHAR for Pharmacology, Toxicology and Pharmaceutics.
- DOCTYPE, "ar" restricts retrieval to articles and excludes conference papers, editorials, notes, and errata indexed as other document types.
- The PUBYEAR > 2009 AND PUBYEAR < 2026 bounds capture records from 2010 to 2025. Because Scopus applies year-level rather than month-level publication-year filtering in this syntax, records published after 31 March 2025, if retrieved, were excluded during screening.
- No separate controlled-vocabulary block was applied.

S1.5 Search-strategy limitations

The database search was designed to retrieve BTC-related economic evidence and therefore combined BTC terms with economic and health-economic outcome terms. Screening, surveillance, prevention, vaccination, and early-detection terms were not included as a separate concept block. As a result, prevention- or screening-focused studies would have been retrieved if they used economic terminology captured by Block 2, or if they were identified through manual reference-list or targeted source checking. This limitation is acknowledged in the manuscript and should be considered when interpreting the prevention and early-detection findings.

The strategies were implemented primarily as free-text, topic-field, or title/abstract/keyword searches, depending on database functionality. Separate controlled-vocabulary blocks, such as MeSH or Emtree headings, were not used. This decision is reported transparently here to support reproducibility.
