# Supplementary material for: Economic Evidence on Biliary Tract Cancer: A Systematic Review
Source: Cancers (Basel). 2026 Jun 25;18(13):2057. doi: 10.3390/cancers18132057 (PMC13360021; doi:10.3390/cancers18132057)
Supplement: Supplementary file 1 [file cancers-18-02057-s001.zip › Supplementary_Material_S2_PRISMA_2020_Checklist_reformatted.pdf]

Supplementary Material S2 - PRISMA 2020 Checklist

Economic evidence on biliary tract cancer: a systematic review

This checklist maps the revised manuscript to the PRISMA 2020 reporting items. Where an item was not applicable because no quantitative meta-analysis, formal certainty assessment, or formal reporting-bias assessment was performed, this is stated explicitly.

Supplementary package: S1, complete database search strategies; S2, PRISMA 2020 checklist; S3, study-level extraction and appraisal, including full-text exclusion summary; S4, cost-currency and conversion notes.

Section and Topic	Item	Checklist Item	Location Where Item Is Reported
**TITLE**	1	Identify the report as a systematic review.	Title page.
**ABSTRACT**	2	See the PRISMA 2020 for Abstracts checklist.	Structured abstract.
**INTRODUCTION**	3	Describe the rationale for the review in the context of existing knowledge.	Introduction.
**INTRODUCTION**	4	Provide an explicit statement of the objective(s) or question(s) the review addresses.	End of Introduction.
**METHODS**	5	Specify the inclusion and exclusion criteria for the review and how studies were grouped for the syntheses.	Section 2.1, Search Strategy and Selection Criteria; Section 2.3, Synthesis and Cost Presentation.
**METHODS**	6	Specify all databases, registers, websites, organisations, reference lists and other sources searched or consulted to identify studies. Specify the date when each source was last searched or consulted.	Section 2.1; Supplementary Material S1. The manuscript states that PubMed/MEDLINE, Embase, Web of Science Core Collection, and Scopus were searched, and that reference lists and selected HTA sources were checked manually.
**METHODS**	7	Present the full search strategies for all databases, registers and websites, including any filters and limits used.	Supplementary Material S1 provides the complete database-specific search strategies, including field tags, date limits, database syntax, document-type filters, and subject-area filters where applicable. S1 also clarifies that screening, surveillance, prevention, and early-detection terms were eligible concepts but were not used as a separate database-search block.
**METHODS**	8	Specify the methods used to decide whether a study met the inclusion criteria of the review, including how many reviewers screened each record and each report retrieved, whether they worked independently, and details of automation tools used in the process.	Section 2.1 states that records were deduplicated, two reviewers independently screened titles/abstracts and assessed full texts, and disagreements were resolved through discussion and consensus. Formal inter-rater agreement statistics were not prospectively recorded.
**METHODS**	9	Specify the methods used to collect data from reports, including how many reviewers collected data from each report, whether they worked independently, any processes for obtaining or confirming data from study investigators, and details of automation tools used in the process.	Section 2.2 states that data were extracted using a standardized form and checked by a second reviewer against source publications. Disagreements or uncertainties were resolved through discussion. Study authors were not routinely contacted. Extracted study-level data are summarized in Supplementary Material S3.
**METHODS**	10a	List and define all outcomes for which data were sought. Specify whether all results compatible with each outcome domain in each study were sought, and if not, the methods used to decide which results to collect.	Section 2.2 defines the economic outcomes extracted, including ICERs, QALYs, life-years, total and incremental costs, willingness-to-pay thresholds, probability of cost-effectiveness, mean or median costs, resource use, hospitalizations, productivity outcomes, and cost drivers.
**METHODS**	10b	List and define all other variables for which data were sought and describe any assumptions made about any missing or unclear information.	Section 2.2 lists extracted study characteristics, including author, year, country or region, study design, population, sample size, intervention/comparator, perspective, model structure, time horizon, discount rate, cost categories, price year and currency, clinical-effectiveness inputs, utility inputs, sensitivity analyses, and authors' conclusions. Supplementary Material S3 provides the study-level extraction and appraisal table.
**METHODS**	11	Specify the methods used to assess risk of bias in the included studies, including details of the tool(s) used, how many reviewers assessed each study, and whether they worked independently.	Section 2.2 explains that CHEERS 2022 was used to assess reporting completeness and the Drummond framework was used to structure methodological appraisal. CHEERS was not treated as a formal risk-of-bias tool. Appraisal was summarized at study level in Supplementary Material S3.
**METHODS**	12	Specify for each outcome the effect measure(s) used in the synthesis or presentation of results.	Sections 2.2 and 2.3. Outcomes were summarized using reported ICERs, cost per QALY, cost per life-year gained, QALYs, life-years, total costs, incremental costs, mean or median cost estimates, per-patient-per-month costs, resource-use measures, and productivity outcomes where available.
**METHODS**	13a	Describe the processes used to decide which studies were eligible for each synthesis.	Section 2.3 explains that studies were grouped narratively by decision context: first-line systemic therapies; second-line and biomarker-driven therapies; screening, prevention, and pathway-adjacent diagnostic strategies; and cost-of-illness or real-world resource-use studies.
**METHODS**	13b	Describe any methods required to prepare the data for presentation or synthesis, such as handling of missing summary statistics, or data conversions.	Section 2.3 explains that monetary values were extracted primarily in original currencies and price contexts. Where review-level conversions were used for orientation, assumptions are documented in Supplementary Material S4.
**METHODS**	13c	Describe any methods used to tabulate or visually display results of individual studies and syntheses.	Section 3.1, Table 1, and Figure 1 in the manuscript; Supplementary Material S3 for study-level extraction and appraisal; Supplementary Material S4 for cost-currency and conversion notes.
**METHODS**	13d	Describe any methods used to synthesize results and provide a rationale for the choice(s). If meta-analysis was performed, describe the model(s), method(s) to identify heterogeneity, and software package(s) used.	Section 2.3 states that meta-analysis was not appropriate because of heterogeneity in study design, interventions, comparators, jurisdictions, perspectives, and outcomes. A narrative synthesis was therefore performed. No statistical meta-analysis was conducted.
**METHODS**	13e	Describe any methods used to explore possible causes of heterogeneity among study results.	Section 2.3 and Discussion. Heterogeneity was explored narratively by intervention class, comparator, country or region, payer perspective, willingness-to-pay threshold, price assumptions, and health-system context.
**METHODS**	13f	Describe any sensitivity analyses conducted to assess robustness of the synthesized results.	Not applicable. No quantitative meta-analysis or review-level sensitivity analysis was performed. Sensitivity analyses reported by individual studies were extracted and summarized where relevant.
**METHODS**	14	Describe any methods used to assess risk of bias due to missing results in a synthesis (arising from reporting biases).	No formal reporting-bias assessment was performed. The possibility of publication bias, selective availability of economic evidence, and incomplete gray-literature coverage is acknowledged in the Limitations section.

Section and Topic	Item	Checklist Item	Location Where Item Is Reported
**METHODS**	15	Describe any methods used to assess certainty (or confidence) in the body of evidence for an outcome.	No formal certainty framework, such as GRADE, was applied. This is acknowledged in the Limitations section. Study-level reporting completeness and methodological credibility were assessed using CHEERS 2022 and the Drummond framework and are summarized in Supplementary Material S3.
**RESULTS**	16a	Describe the results of the search and selection process, from the number of records identified in the search to the number of studies included in the review, ideally using a flow diagram.	Section 3.1 and Figure 1. The manuscript reports records identified, duplicates removed, records screened, full-text reports assessed, full-text exclusions by category, and included studies.
**RESULTS**	16b	Cite studies that might appear to meet the inclusion criteria, but which were excluded, and explain why they were excluded.	Section 3.1 reports full-text exclusion categories and counts. Supplementary Material S3 includes the study-level extraction and appraisal material and summarizes full-text exclusion categories and reasons aligned with the PRISMA flow diagram.
**RESULTS**	17	Cite each included study and present its characteristics.	Table 1 presents the included studies, with reference numbers added to the Author/Year column. Supplementary Material S3 provides additional extracted study-level characteristics and appraisal information.
**RESULTS**	18	Present assessments of risk of bias for each included study.	A formal risk-of-bias score was not applied because the included evidence comprised heterogeneous economic evaluations and descriptive cost/resource-use studies. Reporting completeness and methodological credibility were assessed using CHEERS 2022 and the Drummond framework, respectively. Study-level appraisal results are provided in Supplementary Material S3.
**RESULTS**	19	For all outcomes, present for each study: (a) summary statistics for each group (where appropriate) and (b) an effect estimate and its precision.	Table 1 and Sections 3.2-3.5 present the main economic findings for each included study. Supplementary Material S3 provides additional extracted study-level data. Because most studies were model-based economic evaluations or descriptive cost studies, conventional effect estimates and precision measures were not consistently applicable.
**RESULTS**	20a	For each synthesis, briefly summarise the characteristics and risk of bias among contributing studies.	Sections 3.2-3.5 and Discussion summarize findings by decision context and identify key limitations, including model assumptions, price sensitivity, comparator choice, jurisdictional thresholds, and generalisability. Supplementary Material S3 provides study-level appraisal.
**RESULTS**	20b	Present results of all statistical syntheses conducted. If meta-analysis was done, present for each the summary estimate and its precision and measures of statistical heterogeneity.	Not applicable. No statistical meta-analysis was undertaken.
**RESULTS**	20c	Present results of all investigations of possible causes of heterogeneity among study results.	Sections 3.2-3.5 and Discussion narratively examine heterogeneity by intervention class, jurisdiction, comparator, payer perspective, price assumptions, willingness-to-pay thresholds, and evidence source. No formal subgroup analysis or meta-regression was performed.
**RESULTS**	20d	Present results of all sensitivity analyses conducted to assess the robustness of the synthesized results.	Not applicable at review level. Sensitivity analyses reported within included economic evaluations are summarized narratively and in Supplementary Material S3 where relevant.
**RESULTS**	21	Present assessments of risk of bias due to missing results (arising from reporting biases) for each synthesis assessed.	No formal assessment was performed. Potential publication bias, incomplete gray-literature coverage, and selective availability of economic evidence are discussed in the Limitations section.
**RESULTS**	22	Present assessments of certainty (or confidence) in the body of evidence for each outcome assessed.	No formal certainty assessment was undertaken. The Discussion and Limitations sections describe the overall confidence constraints arising from heterogeneity, model dependence, jurisdictional variation, language restriction, and limited prevention/screening evidence.
**DISCUSSION**	23a	Provide a general interpretation of the results in the context of other evidence.	Discussion.
**DISCUSSION**	23b	Discuss any limitations of the evidence included in the review.	Limitations section.
**DISCUSSION**	23c	Discuss any limitations of the review processes used.	Limitations section, including lack of prospective registration, absence of formal inter-rater agreement statistics, non-fully-duplicate procedures at all stages, English-language restriction, and absence of a separate prevention/screening search block.
**DISCUSSION**	23d	Discuss implications of the results for practice, policy, and future research.	Discussion, Future Directions, and Conclusions.
OTHER INFORMATION	24a	Provide registration information for the review, including register name and registration number, or state that the review was not registered.	Section 2.1 and Limitations state that the review was not prospectively registered.
OTHER INFORMATION	24b	Indicate where the review protocol can be accessed, or state that a protocol was not prepared.	No public protocol is available. The review was not prospectively registered, as stated in Section 2.1 and the Limitations section.
OTHER INFORMATION	24c	Describe and explain any amendments to information provided at registration or in the protocol.	Not applicable because no registered protocol was available.
OTHER INFORMATION	25	Describe sources of financial or non-financial support for the review, and the role of the funders or sponsors in the review.	Funding information is reported in the manuscript Funding section.
OTHER INFORMATION	26	Declare any competing interests of review authors.	Competing interests are reported in the manuscript Conflicts of Interest statement.
OTHER INFORMATION	27	Report which of the following are publicly available and where they can be found: template data collection forms; data extracted from included studies; data used for all analyses; analytic code; any other materials used in the review.	Extracted study-level data and appraisal information are provided in Supplementary Material S3. Full-text exclusions with reasons are provided in Supplementary Material S3. Cost-currency and conversion notes are provided in Supplementary Material S4. The complete database search strategies are provided in Supplementary Material S1. No analytic code was used because no quantitative meta-analysis or statistical model was performed.

Note: Because this review did not perform quantitative meta-analysis, several PRISMA items related to statistical synthesis, review-level sensitivity analyses, reporting-bias assessment, and certainty grading are marked as not applicable or not formally assessed. Study-level reporting completeness and methodological credibility are nevertheless summarized in Supplementary Material S3, while search reproducibility and cost-conversion transparency are supported by Supplementary Materials S1 and S4, respectively.
