# Supplementary material for: Economic Evidence on Biliary Tract Cancer: A Systematic Review
Source: Cancers (Basel). 2026 Jun 25;18(13):2057. doi: 10.3390/cancers18132057 (PMC13360021; doi:10.3390/cancers18132057)
Supplement: Supplementary file 1 [file cancers-18-02057-s001.zip › Supplementary_Material_S3_Study_Extraction_and_Appraisal_reformatted.pdf]

Supplementary Material S3 - Study-Level Extraction and Appraisal

Economic evidence on biliary tract cancer: a systematic review

Overview

This supplement provides the study-level extraction and appraisal information for the 20 studies included in the systematic review. It is intended to support reproducibility, transparency, and traceability between the manuscript, Table 1, and the narrative synthesis.

For each included study, this supplement reports: (i) extracted study characteristics and economic outcomes; (ii) CHEERS 2022 reporting-completeness ratings on items most relevant to economic evidence synthesis; (iii) Drummond methodological-appraisal judgments on the credibility of the analytic approach; (iv) principal limitations identified during appraisal; and (v) the role of the study in the narrative synthesis.

CHEERS 2022 was used as a reporting standard, not as a formal risk-of-bias tool. A Y/P/N rating reflects whether the relevant item was adequately reported, partially or ambiguously reported, or not reported. The Drummond checklist was used to structure methodological appraisal of the analytic choices themselves, including the economic question, comparator, perspective, cost and outcome measurement, valuation, discounting, uncertainty analysis, and interpretation.

Cost-of-illness and descriptive resource-use studies were appraised against the subset of CHEERS 2022 and Drummond items applicable to non-comparative economic analyses. No study was excluded solely on the basis of appraisal findings. Instead, appraisal findings informed the narrative weighting of evidence, with greater interpretive weight given to studies with transparent methods, appropriate comparators, robust clinical inputs, and adequate uncertainty analysis.

Legend

- Y = adequately reported or methodologically sound
- P = partially reported, ambiguous, or incompletely justified
- N = not reported or not addressed
- n/a = not applicable to the study design

Table S3.1a(i) - Extracted study characteristics: source, setting, and design

#	Study	Ref.	Country / setting	Study type
1	Chamberlain 2021	[43]	USA	Cost-of-illness / claims analysis
2	Chen R 2022	[44]	China	Partitioned-survival CEA
3	Chen KA 2024	[45]	Taiwan	Partitioned-survival CEA
4	Choi 2024	[46]	Canada, Ontario	Retrospective administrative-data cost study
5	Chueh 2023	[47]	Taiwan	Partitioned-survival CEA with PSA
6	Darbà and Marsà 2021	[48]	Spain	Retrospective cost-of-illness
7	Jiang C 2025	[49]	China and USA	Cost-utility CEA
8	Kashiwa and Maeda 2024	[50]	Japan	Comparative CEA
9	Laopachee 2023	[51]	Thailand	Decision-tree CEA
10	Lundgren 2020	[52]	Sweden	Decision-tree CEA
11	Luo X 2024	[53]	China and USA	Markov CEA
12	Olthof 2018	[54]	Netherlands	Retrospective cost analysis
13	Parasuraman 2023	[55]	USA	Retrospective cost and productivity analysis
14	Roth and Carlson 2012	[56]	USA	Markov CEA
15	Tsukiyama 2017	[57]	Japan	Markov CEA
16	Wadhwa 2017	[58]	USA	Retrospective inpatient cost analysis
17	Wang 2024	[59]	USA	Retrospective resource-use and cost study
18	Ye 2023	[37]	USA and China	Markov CEA
19	Zhao 2023	[60]	China	Partitioned-survival CEA
20	Zheng 2023	[61]	China	Cost-utility analysis

Table S3.1a(ii) - Extracted study characteristics: population, perspective, and comparator

#	Study	Population	Perspective	Intervention / comparator
1	Chamberlain 2021	Advanced cholangiocarcinoma	US payer	Descriptive; no formal comparator
2	Chen R 2022	Advanced BTC	Chinese payer / National Health Insurance	XELOX vs GEMOX
3	Chen KA 2024	Previously treated IDH1-mutant advanced iCCA	Taiwanese NHI payer	Ivosidenib vs mFOLFOX or 5-FU/LV
4	Choi 2024	BTC-related hospitalizations	Hospital / health-system perspective	Descriptive; by BTC subtype
5	Chueh 2023	FGFR2-fusion advanced iCCA	Taiwanese NHIA payer	Pemigatinib vs 5-FU/LV or mFOLFOX
6	Darbà and Marsà 2021	Intrahepatic cholangiocarcinoma hospital admissions	Hospital perspective	Descriptive
7	Jiang C 2025	First-line advanced BTC	Chinese and US payer perspectives	Pembrolizumab + GemCis vs GemCis
8	Kashiwa and Maeda 2024	First-line advanced BTC	Japanese payer	GCS, durvalumab + GemCis, pembrolizumab + GemCis vs GemCis
9	Laopachee 2023	High-risk adults in CCA-endemic area	Societal perspective	Ultrasound surveillance vs no screening
10	Lundgren 2020	Cholecystectomy for benign	Swedish healthcare payer	Routine vs selective gallbladder

#	Study	Population	Perspective	Intervention / comparator
		disease		histopathology strategies
11	Luo X 2024	First-line advanced BTC	Chinese and US payer perspectives	Pembrolizumab + GemCis vs GemCis
12	Olthof 2018	Cholecystectomy for benign gallbladder disease	Societal perspective	Routine pathology and follow-up vs selective approach
13	Parasuraman 2023	Working-age patients with cholangiocarcinoma	Payer and societal perspectives	Descriptive; by subtype
14	Roth and Carlson 2012	Advanced BTC	Societal perspective	GemCis vs gemcitabine
15	Tsukiyama 2017	Advanced BTC	Japanese healthcare payer	GemCis vs gemcitabine
16	Wadhwa 2017	Cholangiocarcinoma admissions	Hospital charges perspective	Descriptive
17	Wang 2024	Advanced BTC	Payer perspective	Descriptive; by treatment line
18	Ye 2023	First-line advanced BTC	US and Chinese payer perspectives	Durvalumab + GemCis vs GemCis
19	Zhao 2023	First-line advanced BTC	Chinese payer	Durvalumab + GemCis vs GemCis
20	Zheng 2023	First-line advanced BTC	Chinese payer	Pembrolizumab + GemCis vs GemCis

Table S3.1b(i) - Economic findings: model/data source and cost context

#	Study	Model or data source	Time horizon	Discount rate	Currency / price year
1	Chamberlain 2021	Optum Clinformatics claims	Follow-up as reported	n/a	USD; price year as reported
2	Chen R 2022	Trial-informed model	As reported	As reported	CNY / USD as reported
3	Chen KA 2024	ClarIDHy-informed model	As reported	As reported	NT$; price year as reported
4	Choi 2024	GEMINI hospital data	2016-2021	n/a	CAD; price year as reported
5	Chueh 2023	FIGHT-202-informed model	As reported	As reported	NT$; price year as reported
6	Darbà and Marsà 2021	National hospital discharge database	2000-2018	n/a	EUR; price year as reported
7	Jiang C 2025	KEYNOTE-966-informed model	As reported	As reported	USD / CNY as reported
8	Kashiwa and Maeda 2024	Trial-informed model	10 years	As reported	JPY; price year as reported
9	Laopachee 2023	Screening cohort / decision tree	As reported	As reported	THB; price year as reported
10	Lundgren 2020	Registry-informed decision tree	As reported	As reported	EUR; price year as reported
11	Luo X 2024	KEYNOTE-966-informed model	As reported	As reported	USD / CNY as reported
12	Olthof 2018	Single-centre data	As reported	n/a or as reported	EUR; price year as reported
13	Parasuraman 2023	OptumHealth claims	As reported	n/a	USD; price year as reported
14	Roth and Carlson 2012	ABC-02-informed model	As reported	As reported	USD; price year as reported
15	Tsukiyama 2017	BT-22-informed model	As reported	As reported	JPY; price year as reported
16	Wadhwa 2017	National Inpatient Sample	1997-2012	n/a	USD; price year as reported
17	Wang 2024	Linked EHR/claims data	As reported	n/a	USD; price year as reported
18	Ye 2023	TOPAZ-1-informed model	As reported	As reported	USD / CNY as reported
19	Zhao 2023	TOPAZ-1-informed model	As reported	As reported	CNY / USD as reported
20	Zheng 2023	KEYNOTE-966-informed model	As reported	As reported	USD / CNY as reported

Table S3.1b(ii) - Economic findings: outcome, uncertainty, and role in synthesis

#	Study	Main economic outcome	Key uncertainty or sensitivity findings	Role in synthesis
1	Chamberlain 2021	High per-patient-per-month costs; medical services were the main cost component	Generalisability limited to insured US population	Real-world economic burden
2	Chen R 2022	XELOX dominated GEMOX in the base case	Sensitive to costs, utilities, and survival assumptions	First-line chemotherapy
3	Chen KA 2024	ICERs exceeded Taiwanese WTP threshold	Price reductions of approximately 50-60% were needed	Biomarker-driven therapy
4	Choi 2024	Hospital burden and costs increased over time	Limited to hospital-based costs	Real-world hospital burden
5	Chueh 2023	Pemigatinib was not cost-effective at assumed price	Drug price was a major driver; substantial price reduction improved value	Biomarker-driven therapy
6	Darbà and Marsà 2021	Direct medical costs remained substantial	Limited to inpatient care	Real-world hospital burden
7	Jiang C 2025	Pembrolizumab combination was not cost-effective in either setting	Pembrolizumab price was a major driver	First-line immunotherapy

#	Study	Main economic outcome	Key uncertainty or sensitivity findings	Role in synthesis
8	Kashiwa and Maeda 2024	GCS was cost-effective; immunotherapy combinations were not	Results sensitive to drug prices and survival assumptions	First-line chemotherapy and immunotherapy
9	Laopachee 2023	Ultrasound surveillance was cost-effective in endemic setting	Context-specific to high-risk endemic population	Screening and early detection
10	Lundgren 2020	Selective histopathology could reduce costs with limited loss of benefit	Applies to low-risk benign-disease pathway	Pathway-adjacent diagnostic evidence
11	Luo X 2024	Pembrolizumab combination was not cost-effective	Drug price and utility assumptions influenced ICERs	First-line immunotherapy
12	Olthof 2018	Selective pathology could reduce unnecessary spending	Single-centre pathway-adjacent evidence	Pathway-adjacent diagnostic evidence
13	Parasuraman 2023	High direct costs and productivity losses	Limited to commercially insured working-age population	Real-world burden and productivity loss
14	Roth and Carlson 2012	GemCis was cost-effective at US WTP thresholds	Sensitive to survival, utilities, and progression costs	First-line chemotherapy
15	Tsukiyama 2017	GemCis was not cost-effective at Japanese threshold	Low probability of cost-effectiveness at threshold used	First-line chemotherapy
16	Wadhwa 2017	Admissions and hospital charges increased over time	Inpatient-only and charge-based estimates	Real-world hospital burden
17	Wang 2024	Costs increased across later treatment lines	Limited by real-world data completeness	Real-world treatment-line costs
18	Ye 2023	Durvalumab combination was not cost-effective	Durvalumab price was a key driver	First-line immunotherapy
19	Zhao 2023	Durvalumab combination was not cost-effective, even with assistance	Large price reduction required	First-line immunotherapy
20	Zheng 2023	Pembrolizumab combination was not cost-effective	Pembrolizumab price, PD utility, and subsequent treatment costs were influential	First-line immunotherapy

Table S3.2 - CHEERS 2022 reporting-completeness ratings

CHEERS 2022 items were condensed into the following domains for readability. These ratings assess reporting completeness only; they do not imply that the reported methodological choices were necessarily correct. The item summary is provided in Table S3.6.

- C1 Title and abstract identify economic evaluation (items 1-2)
- C2 Background, objectives, population, setting (items 3, 5, 6)
- C3 Comparators, perspective, time horizon (items 7-9)
- C4 Discount rate (item 10)
- C5 Outcome selection, measurement, valuation (items 11-13)
- C6 Resource and cost measurement, valuation (item 14)
- C7 Currency, price date, conversion (item 15)
- C8 Model rationale and structure (items 16-17)
- C9 Heterogeneity (item 18)
- C10 Distributional effects (item 19)
- C11 Characterising uncertainty (item 20)
- C12 Engagement with patients/public (item 21)
- C13 Parameters and main results (items 22-23)
- C14 Effect of uncertainty (item 24)
- C15 Findings, limitations, generalisability (item 26)
- C16 Funding and conflicts (items 27-28)

Table S3.2a - CHEERS ratings, domains C1-C8

#	Study	Design	C1	C2	C3	C4	C5	C6	C7	C8
1	Chamberlain 2021	Cost-of-illness (US claims)	Y	Y	P	n/a	Y	Y	P	n/a
2	Chen R 2022	Partitioned-survival CEA (China)	Y	Y	Y	Y	Y	Y	P	Y
3	Chen KA 2024	Partitioned-survival CEA (Taiwan)	Y	Y	Y	Y	Y	Y	P	Y
4	Choi 2024	Retrospective cost cohort (Canada)	Y	Y	P	n/a	Y	Y	Y	n/a
5	Chueh 2023	Partitioned-survival CEA + PSA (Taiwan)	Y	Y	Y	Y	Y	Y	P	Y
6	Darbà 2021	Retrospective cost-of-illness (Spain)	Y	Y	P	n/a	Y	Y	P	n/a
7	Jiang C 2025	Cost-utility CEA (China & USA)	Y	Y	Y	Y	Y	Y	P	Y
8	Kashiwa 2024	Cost-effectiveness CEA (Japan)	Y	Y	Y	Y	Y	Y	P	Y
9	Laopachee 2023	Decision-tree CEA, screening (Thailand)	Y	Y	Y	Y	Y	Y	Y	Y
10	Lundgren 2020	Decision-tree CEA, pathology (Sweden)	Y	Y	Y	Y	Y	Y	P	Y
11	Luo X 2024	Markov CEA (China & USA)	Y	Y	Y	Y	Y	Y	P	Y
12	Olthof 2018	Retrospective cost analysis (NL)	Y	Y	P	n/a	Y	Y	P	n/a
13	Parasuraman 2023	Retrospective cost + productivity (US)	Y	Y	P	n/a	Y	Y	Y	n/a
14	Roth & Carlson 2012	Markov CEA (US)	Y	Y	Y	Y	Y	Y	P	Y
15	Tsukiyama 2017	Markov CEA, BT-22 trial-based (Japan)	Y	Y	Y	Y	Y	Y	P	Y
16	Wadhwa 2017	Retrospective cost-of-illness (US NIS)	Y	Y	P	n/a	Y	Y	P	n/a
17	Wang 2024	Retrospective cost cohort (US)	Y	Y	P	n/a	Y	Y	P	n/a
18	Ye 2023	Markov CEA (US & China)	Y	Y	Y	Y	Y	Y	P	Y
19	Zhao 2023	3-state partitioned-survival CEA (China)	Y	Y	Y	Y	Y	Y	P	Y
20	Zheng 2023	Cost-utility analysis, Markov-like (China)	Y	Y	Y	Y	Y	Y	P	Y

Table S3.2b - CHEERS ratings, domains C9-C16

#	Study	C9	C10	C11	C12	C13	C14	C15	C16
1	Chamberlain 2021	P	n/a	n/a	N	Y	n/a	Y	Y
2	Chen R 2022	P	n/a	Y	N	Y	Y	Y	Y
3	Chen KA 2024	P	n/a	Y	N	Y	Y	Y	Y
4	Choi 2024	P	n/a	n/a	N	Y	n/a	Y	Y
5	Chueh 2023	Y	n/a	Y	N	Y	Y	Y	Y
6	Darbà 2021	P	n/a	n/a	N	Y	n/a	Y	Y
7	Jiang C 2025	Y	n/a	Y	N	Y	Y	Y	Y
8	Kashiwa 2024	P	n/a	Y	N	Y	Y	Y	Y
9	Laopachee 2023	P	n/a	Y	N	Y	Y	Y	Y
10	Lundgren 2020	P	n/a	Y	N	Y	Y	Y	Y
11	Luo X 2024	Y	n/a	Y	N	Y	Y	Y	Y
12	Olthof 2018	P	n/a	n/a	N	Y	n/a	Y	Y
13	Parasuraman 2023	Y	n/a	n/a	N	Y	n/a	Y	Y
14	Roth & Carlson 2012	Y	n/a	Y	N	Y	Y	Y	Y
15	Tsukiyama 2017	P	n/a	Y	N	Y	Y	Y	Y
16	Wadhwa 2017	P	n/a	n/a	N	Y	n/a	Y	Y
17	Wang 2024	Y	n/a	n/a	N	Y	n/a	Y	Y
18	Ye 2023	P	n/a	Y	N	Y	Y	Y	Y
19	Zhao 2023	P	n/a	Y	N	Y	Y	Y	Y
20	Zheng 2023	Y	n/a	Y	N	Y	Y	Y	Y

Notes for Table S3.2

C4, discount rate. For model-based cost-effectiveness and cost-utility studies, reported discount rates were compared with jurisdictional pharmacoeconomic guidance where this was identifiable. Studies were rated Y when discounting was clearly reported and broadly consistent with jurisdictional or commonly used health-economic guidance; P when reporting was incomplete or the rationale was unclear; and n/a for descriptive cost-of-illness or resource-use studies where discounting was not applicable. The rating reflects reporting and methodological adequacy within the context of each source study, not an independent re-estimation of costs or outcomes.

C7, currency, price date, and conversion. Most studies were rated P because the price year and in-paper conversion approach were reported but not always with the level of detail required by CHEERS 2022 item 15, particularly annual-average exchange rates and inflation index sources. The review-level approach to currency and conversion is documented separately in Supplementary Material S4.

C9, heterogeneity. Subgroup or scenario analyses were rated Y when the source manuscript performed probabilistic, deterministic, subgroup, or scenario analyses that allowed some assessment of heterogeneity or variation in results. Other model-based studies were rated P. Among real-world cost studies, Y was assigned when the study explicitly stratified by key clinical or utilization groups.

C10, distributional effects. None of the twenty studies explicitly characterised distributional effects across subpopulations as a distributional cost-effectiveness analysis or equivalent equity analysis. This item was therefore marked n/a where the study did not claim to address distributional effects.

C11, uncertainty characterisation. Model-based CEAs were rated Y when they reported probabilistic sensitivity analysis, deterministic sensitivity analysis, or scenario analysis. Descriptive resource-use studies were generally rated n/a unless formal uncertainty or variability analysis was applicable to their design.

C12, patient/public engagement. None of the twenty studies reported formal patient or public engagement in study design or interpretation; this domain was therefore rated N across included studies.

Funding sources and conflicts of interest were reviewed for each included study against CHEERS 2022 items 27-28. The purpose was to assess transparency of reporting, not to exclude studies on the basis of funding source. Where a study reported industry funding, industry-employed authors, or other relevant relationships, this was considered during narrative interpretation alongside methodological features such as comparator choice, price inputs, uncertainty analysis, and transparency of assumptions. No included study was excluded solely because of its funding source or declared conflicts.

Table S3.3 - Funding and conflict-of-interest transparency

#	Study	Ref.	Funding source as reported	Conflicts of interest as reported	Appraisal note
1	Chamberlain 2021	[43]	As reported in source publication	As reported in source publication	Reviewed for reporting transparency; not used as an exclusion criterion.
2	Chen R 2022	[44]	As reported in source publication	As reported in source publication	Reviewed for reporting transparency; not used as an exclusion criterion.
3	Chen KA 2024	[45]	As reported in source publication	As reported in source publication	Reviewed for reporting transparency; not used as an exclusion criterion.
4	Choi 2024	[46]	As reported in source publication	As reported in source publication	Reviewed for reporting transparency; not used as an exclusion criterion.
5	Chueh 2023	[47]	As reported in source publication	As reported in source publication	Reviewed for reporting transparency; not used as an exclusion criterion.
6	Darbà and Marsà 2021	[48]	As reported in source publication	As reported in source publication	Reviewed for reporting transparency; not used as an exclusion criterion.
7	Jiang C 2025	[49]	As reported in source publication	As reported in source publication	Reviewed for reporting transparency; not used as an exclusion criterion.
8	Kashiwa and Maeda 2024	[50]	As reported in source publication	As reported in source publication	Reviewed for reporting transparency; not used as an exclusion criterion.
9	Laopachee 2023	[51]	As reported in source publication	As reported in source publication	Reviewed for reporting transparency; not used as an exclusion criterion.
10	Lundgren 2020	[52]	As reported in source publication	As reported in source publication	Reviewed for reporting transparency; not used as an exclusion criterion.
11	Luo X 2024	[53]	As reported in source publication	As reported in source publication	Reviewed for reporting transparency; not used as an exclusion criterion.
12	Olthof 2018	[54]	As reported in source publication	As reported in source publication	Reviewed for reporting transparency;

#	Study	Ref.	Funding source as reported	Conflicts of interest as reported	Appraisal note
					not used as an exclusion criterion.
13	Parasuraman 2023	[55]	As reported in source publication	As reported in source publication	Reviewed for reporting transparency; not used as an exclusion criterion.
14	Roth and Carlson 2012	[56]	As reported in source publication	As reported in source publication	Reviewed for reporting transparency; not used as an exclusion criterion.
15	Tsukiyama 2017	[57]	As reported in source publication	As reported in source publication	Reviewed for reporting transparency; not used as an exclusion criterion.
16	Wadhwa 2017	[58]	As reported in source publication	As reported in source publication	Reviewed for reporting transparency; not used as an exclusion criterion.
17	Wang 2024	[59]	As reported in source publication	As reported in source publication	Reviewed for reporting transparency; not used as an exclusion criterion.
18	Ye 2023	[37]	As reported in source publication	As reported in source publication	Reviewed for reporting transparency; not used as an exclusion criterion.
19	Zhao 2023	[60]	As reported in source publication	As reported in source publication	Reviewed for reporting transparency; not used as an exclusion criterion.
20	Zheng 2023	[61]	As reported in source publication	As reported in source publication	Reviewed for reporting transparency; not used as an exclusion criterion.

Table S3.4 - Drummond methodological-credibility appraisal

The Drummond ten-point checklist was used to appraise methodological credibility. For non-comparative cost-of-illness and resource-use studies, comparator-specific items, including comprehensive description of alternatives and incremental analysis, were marked n/a. The net judgement column summarizes how the appraisal influenced the narrative synthesis.

Table S3.4a - Drummond ratings

#	Study	D1	D2	D3	D4	D5	D6	D7	D8	D9	D10
1	Chamberlain 2021	Y	n/a	n/a	Y	Y	Y	n/a	n/a	P	Y
2	Chen R 2022	Y	Y	Y	Y	Y	Y	Y	Y	Y	Y
3	Chen KA 2024	Y	Y	Y	Y	Y	Y	Y	Y	Y	Y
4	Choi 2024	Y	n/a	n/a	Y	Y	Y	n/a	n/a	Y	Y
5	Chueh 2023	Y	Y	Y	Y	Y	Y	Y	Y	Y	Y
6	Darbà 2021	Y	n/a	n/a	Y	Y	Y	n/a	n/a	P	Y
7	Jiang C 2025	Y	Y	Y	Y	Y	Y	Y	Y	Y	Y
8	Kashiwa 2024	Y	Y	Y	Y	Y	Y	Y	Y	P	Y
9	Laopachee 2023	Y	Y	Y	Y	Y	Y	Y	Y	Y	Y
10	Lundgren 2020	Y	Y	Y	Y	Y	Y	Y	Y	P	Y
11	Luo X 2024	Y	Y	Y	Y	Y	Y	Y	Y	Y	Y
12	Olthof 2018	Y	n/a	n/a	Y	Y	Y	n/a	n/a	P	Y
13	Parasuraman 2023	Y	n/a	n/a	Y	Y	Y	n/a	n/a	Y	Y
14	Roth & Carlson 2012	Y	Y	Y	Y	Y	Y	Y	Y	Y	Y
15	Tsukiyama 2017	Y	Y	Y	Y	Y	Y	Y	Y	P	Y
16	Wadhwa 2017	Y	n/a	n/a	Y	Y	Y	n/a	n/a	P	Y
17	Wang 2024	Y	n/a	n/a	Y	Y	Y	n/a	n/a	Y	Y
18	Ye 2023	Y	Y	Y	Y	Y	Y	Y	Y	P	Y
19	Zhao 2023	Y	Y	Y	Y	Y	Y	Y	Y	Y	Y
20	Zheng 2023	Y	Y	Y	Y	Y	Y	Y	Y	Y	Y

Table S3.4b - Drummond net methodological judgement

#	Study	Net methodological judgement
1	Chamberlain 2021	Credible descriptive cost-of-illness; payer-perspective US claims; generalisability limited to insured US population.
2	Chen R 2022	Standard partitioned-survival CEA based on a randomized non-inferiority trial; generalisability bounded by Chinese NHIA setting.
3	Chen KA 2024	Trial-anchored CEA with PSA; explicit price-reduction threshold for cost-effectiveness; Taiwan-specific WTP.
4	Choi 2024	Administrative-data cost study with explicit inflation adjustment; LOS and mortality interpretation appropriate.
5	Chueh 2023	Trial-anchored CEA with PSA; explicit price reduction needed for cost-effectiveness; Taiwan WTP framing.
6	Darbà 2021	Hospital-discharge-database cost-of-illness; cost plateau and mortality reported; perspective is hospital-level, not full societal.
7	Jiang C 2025	Two-jurisdiction Markov CEA, KEYNOTE-966 anchored; pembrolizumab price identified as the dominant input.
8	Kashiwa 2024	Three-arm CEA distinguishing GCS triplet vs immunotherapy combinations; Japanese payer perspective.
9	Laopachee 2023	Decision-tree CEA in liver-fluke-endemic Thai cohort; ICER below local WTP; generalisability strictly bounded to comparable settings.
10	Lundgren 2020	Decision-tree CEA of pathology strategies; selective strategy cost-saving; outcome valuation hinges on missed-cancer probability inputs.
11	Luo X 2024	Two-jurisdiction Markov CEA; both ICERs above WTP; pembrolizumab price-driven.
12	Olthof 2018	Single-centre cost analysis; unnecessary spend identified with very low estimated missed-cancer risk; external generalisability limited.
13	Parasuraman 2023	Combined direct-cost and indirect-cost analysis; subgroup by anatomical subtype; payer and societal perspectives.
14	Roth & Carlson 2012	Foundational US CEA establishing GemCis at approximately US$59,480/QALY; price year is historical and influences interpretation.
15	Tsukiyama 2017	BT-22-anchored Japanese CEA; high ICER versus Japanese threshold; deterministic sensitivity analysis.
16	Wadhwa 2017	US-NIS descriptive cost trend; inflation-adjusted; appropriate for trend interpretation, not comparative judgement.
17	Wang 2024	Treatment-line stratified resource use; integrated EHR and claims; not a CEA.

#	Study	Net methodological judgement
18	Ye 2023	Two-jurisdiction Markov CEA; ICERs above WTP; durvalumab price the dominant driver.
19	Zhao 2023	TOPAZ-1-anchored Chinese CEA; large price reduction required for cost-effectiveness under Chinese WTP; charity-assistance scenario reported.
20	Zheng 2023	KEYNOTE-966-anchored Chinese CEA; well above local WTP; pembrolizumab price and progressed-disease utility influential.

Drummond rating notes

D7, discounting. Model-based economic evaluations were rated Y when discounting was clearly reported and appropriate for the time horizon and jurisdictional context. Cost-of-illness and descriptive resource-use studies were marked n/a when discounting was not applicable to the analysis.

D9, uncertainty allowance. Probabilistic sensitivity analysis plus deterministic sensitivity analysis was generally rated Y. Deterministic-only, scenario-only, or limited uncertainty reporting was rated P. Descriptive cost studies were rated Y when they reported variability, confidence intervals, interquartile ranges, or meaningful subgroup ranges, and P when they did not.

Table S3.5 - Principal limitations and weight in narrative synthesis

#	Study	Principal limitations	Weight in narrative synthesis	Methodological notes
1	Chamberlain 2021	Insured US-only population; claims data miss out-of-pocket spending; no formal comparator; descriptive only.	Supportive for real-world resource-use synthesis; not used for cross-jurisdiction comparison.	Cost-of-illness; discounting not applicable.
2	Chen R 2022	Single-jurisdiction China model; trial-derived effectiveness may have limited external validity beyond NHIA context.	Primary evidence for XELOX dominating GEMOX in the Chinese first-line context.	Discount rate as reported by source study.
3	Chen KA 2024	Hypothetical IDH1-mutant cohort; reliant on ClarIDHy-derived efficacy; Taiwan WTP framing.	Primary evidence for ivosidenib not being cost-effective at list price in Taiwan.	Discount rate as reported by source study.
4	Choi 2024	Ontario-only; hospital perspective; no out-of-hospital costs.	Primary evidence for BTC hospital burden in Canada.	Cost cohort; discounting not applicable.
5	Chueh 2023	Hypothetical FGFR2-fusion cohort based on phase II evidence; Taiwan-specific.	Primary evidence for pemigatinib not being cost-effective at the hypothesized price.	Discount rate as reported by source study.
6	Darbà 2021	Hospital perspective only; iCCA only; pre-2019 data.	Supportive evidence for rising iCCA hospital burden in Spain.	Cost-of-illness; discounting not applicable.
7	Jiang C 2025	Two-jurisdiction modelling; reliant on KEYNOTE-966 efficacy; cost dominated by pembrolizumab price.	Primary evidence for pembrolizumab + GemCis not being cost-effective in US and China settings.	Discount rates as reported by source study.
8	Kashiwa 2024	Cross-trial comparisons across KHBO1401-MITSUBA, TOPAZ-1, and KEYNOTE-966 are subject to indirect-comparison bias.	Primary evidence that GCS may be cost-effective in Japan, while immunotherapy combinations were not.	Discount rate as reported by source study.
9	Laopachee 2023	Single high-incidence Thai endemic setting; generalisability limited to comparable settings.	Primary and only included BTC-specific screening study.	Discount rate as reported by source study.
10	Lundgren 2020	Sensitive to missed-cancer probability assumptions; Swedish payer setting.	Primary evidence for selective gallbladder histopathology as pathway-adjacent evidence.	Discount rate as reported by source study.
11	Luo X 2024	Two-jurisdiction Markov model; KEYNOTE-966 anchored; pembrolizumab price dominant.	Primary evidence concordant with Jiang and Zheng on pembrolizumab non-cost-effectiveness.	Discount rates as reported by source study.
12	Olthof 2018	Single Dutch centre; macroscopic-selective policy generalisability bounded.	Primary pathway-adjacent evidence on gallbladder pathology.	Cost analysis; discounting not applicable.
13	Parasuraman 2023	Working-age commercially insured US population; productivity-loss valuation method-specific.	Primary evidence for direct and indirect cost burden.	Cost analysis; discounting not applicable.
14	Roth and Carlson 2012	Historical US price year; ABC-02-anchored efficacy; current-price interpretation requires caution.	Primary foundational GemCis CEA for US setting.	Discount rate as reported by source study.
15	Tsukiyama 2017	BT-22-anchored Japanese CEA; sensitivity analysis more limited than newer models.	Primary evidence showing jurisdictional contrast for GemCis.	Discount rate as reported by source study.
16	Wadhwa 2017	Older US discharge data; charges rather than costs; trend analysis only.	Supportive evidence for admissions and hospital-charge burden.	Cost-of-illness; discounting not applicable.
17	Wang 2024	US-only EHR/claims; treatment-line stratification dependent on coding completeness.	Primary evidence for costs across first, second, and third treatment lines.	Cost cohort; discounting not applicable.
18	Ye 2023	Two-jurisdiction model; TOPAZ-1 anchored; durvalumab price dominant.	Primary evidence for durvalumab + GemCis not being cost-effective in US/China settings.	Discount rates as reported by source study.
19	Zhao 2023	TOPAZ-1-anchored Chinese CEA; charity-assistance scenario context-specific.	Primary evidence concordant with Ye for durvalumab in China.	Discount rate as reported by source study.
20	Zheng 2023	KEYNOTE-966-anchored Chinese CEA; pembrolizumab price and PD utility influential.	Primary evidence concordant with Luo and Jiang for pembrolizumab in China.	Discount rate as reported by source study.

Table S3.6 - Summary of CHEERS 2022 and Drummond items used for appraisal

Domain	Summary item used in this supplement
CHEERS 1-2	Title and abstract identify the study as an economic evaluation and provide a structured summary.
CHEERS 3, 5, 6	Background, objectives, population, setting, and location are clearly described.
CHEERS 7-9	Comparators, perspective, and time horizon are reported and justified.
CHEERS 10	Discount rate is reported and justified where applicable.
CHEERS 11-13	Outcome selection, measurement, and valuation are described.
CHEERS 14-15	Resource/cost measurement, valuation, currency, price date, and conversion are reported.
CHEERS 16-17	Model rationale, structure, assumptions, and analytics are described where modelling is used.
CHEERS 18-21	Heterogeneity, distributional effects, uncertainty, and patient/public engagement are reported where applicable.
CHEERS 22-24	Study parameters, main results, and effect of uncertainty are reported.
CHEERS 26-28	Findings, limitations, generalisability, funding, and conflicts of interest are reported.

Domain	Summary item used in this supplement
Drummond D1	Was a well-defined question posed in answerable form?
Drummond D2	Was a comprehensive description of competing alternatives given?
Drummond D3	Was the effectiveness of the programmes or services established?
Drummond D4	Were all important and relevant costs and consequences identified?
Drummond D5	Were costs and consequences measured accurately in appropriate units?
Drummond D6	Were costs and consequences valued credibly?
Drummond D7	Were costs and consequences adjusted for differential timing?
Drummond D8	Was an incremental analysis performed?
Drummond D9	Was allowance made for uncertainty?
Drummond D10	Did presentation and discussion include issues of concern to users?

Table S3.7 - Full-text exclusion categories and reasons

This table summarizes the full-text exclusion categories corresponding to the PRISMA flow diagram and the manuscript Results section. The table is included to make the exclusion logic auditable and to align Supplementary Material S3 with PRISMA 2020 item 16b. If a record-level screening log is available, this table should be supplemented with the individual bibliographic records excluded at full-text stage.

Exclusion category	n	Specific reason used for exclusion
Not BTC-specific	12	The report did not provide biliary tract cancer-specific economic results, or BTC findings could not be isolated from a broader cancer or gastrointestinal cohort.
No original economic data	8	The report discussed costs, value, or policy context but did not include original cost, resource-use, cost-effectiveness, cost-utility, cost-benefit, or cost-of-illness data.
Duplicate or superseded analysis	10	The report duplicated an included analysis, used the same underlying dataset without additional relevant economic information, or was superseded by a more complete peer-reviewed publication.
Conference abstract without sufficient data	8	The record was available only as a conference abstract or short meeting report and did not contain sufficient extractable methods, cost inputs, outcomes, or uncertainty information.
Not available in English	5	The full text was not available in English at eligibility assessment and did not provide sufficient extractable English-language economic data.
Other methodological limitations precluding meaningful synthesis	9	The report appeared potentially relevant at screening but could not be synthesized because of insufficient BTC-specific attribution, unclear or non-reproducible economic methods, missing denominator or comparator information, non-extractable monetary outcomes, or otherwise inadequate information to support inclusion.
Total full-text exclusions	52	Matches the total number of reports excluded after full-text assessment in Figure 1 and the Results section.

Note: This table summarizes the exclusion logic using the categories retained in the manuscript. It does not create additional evidence used in the synthesis. A record-level excluded-study log should be used if the original screening database is available.

References for supplementary appraisal guidance

Studies cited in this supplement use the same reference numbers as the main manuscript reference list. Full bibliographic details are provided in the manuscript reference list.

This supplement cites CHEERS 2022 [41] and the Drummond framework [42] as appraisal guidance; both references are included in the main manuscript reference list.

Pharmacoeconomic guideline citations used to contextualize discount-rate reporting are available in the corresponding source publications and national pharmacoeconomic guidance. They were used only to support appraisal of reporting and methodological plausibility, not to re-estimate study results.
