# Supplementary material for: Economic Evidence on Biliary Tract Cancer: A Systematic Review
Source: Cancers (Basel). 2026 Jun 25;18(13):2057. doi: 10.3390/cancers18132057 (PMC13360021; doi:10.3390/cancers18132057)
Supplement: Supplementary file 1 [file cancers-18-02057-s001.zip › Supplementary_Material_S4_Cost_Currency_and_Conversion_Notes_reformatted.pdf]

Supplementary Material S4 - Cost-Currency and Conversion Notes

Economic evidence on biliary tract cancer: a systematic review

Overview

This supplement documents the monetary values used in the manuscript and Table 1 where currency, price year, inflation adjustment, or USD-equivalent orientation values require additional explanation. It reports the original currency and price year as stated in each source study, the inflation index used where review-level adjustment was applied, and the exchange rate used to generate USD-equivalent orientation values.

The purpose of this supplement is transparency and auditability. USD-equivalent values are provided only to aid interpretation across studies; they should not be interpreted as fully standardized values or as a basis for ranking interventions across jurisdictions.

This supplement supports the manuscript sections on Synthesis and Cost Presentation, Table 1, Figure 2, and the Limitations section. It also supports PRISMA 2020 transparency expectations for synthesis methods and CHEERS 2022 items on resource and cost measurement, price date, currency, and conversion [40,41].

S4.1 Methodology

Reference price year

The reference price year used for review-level orientation values is 2024. This year was selected because it was the most recent year for which annual consumer-price-index values and annual-average exchange rates were available across the countries represented in the included evidence base. Where a study's results are quoted in the original currency, the original price year reported by the study is preserved alongside any USD-equivalent orientation value.

Inflation indices

Inflation adjustment from a study's reported price year to 2024 used country-specific consumer price indices from public sources. The default cross-country source was the World Bank FP.CPI.TOTL annual consumer price index series [76]. Taiwan and Thailand required separate national sources, as noted below [78,79].

Table S4.1 - Inflation indices used for review-level adjustments

Country / region	Index series	Source agency
United States	FP.CPI.TOTL, annual consumer price index	World Bank Open Data [76]
Canada	FP.CPI.TOTL, annual consumer price index	World Bank Open Data [76]
Spain	FP.CPI.TOTL, annual consumer price index	World Bank Open Data [76]
Sweden	FP.CPI.TOTL, annual consumer price index	World Bank Open Data [76]
Netherlands	FP.CPI.TOTL, annual consumer price index	World Bank Open Data [76]
Japan	FP.CPI.TOTL, annual consumer price index	World Bank Open Data [76]
China, mainland	FP.CPI.TOTL, annual consumer price index	World Bank Open Data [76]
Thailand	Annual headline CPI percentage change	Bank of Thailand / Ministry of Commerce [79]
Taiwan	Annual CPI series	Directorate-General of Budget, Accounting and Statistics, Taiwan [78]

The World Bank FP.CPI.TOTL series was used as the cross-country default because it is available on a comparable basis for most countries in the review. Healthcare-specific indices were not substituted because the included studies did not consistently use healthcare-specific deflators and because general CPI provides a transparent cross-study convention for an evidence synthesis. For Taiwan, the DGBAS national CPI series was used. For Thailand, headline CPI changes from the Bank of Thailand / Ministry of Commerce were used because these were considered more appropriate for the relevant adjustment window.

Exchange rates

USD-equivalent orientation values were generated using 2024 annual-average exchange rates from the U.S. Federal Reserve G.5A annual release [77]. Rates are expressed as foreign currency units per one U.S. dollar, except for the Euro convention, which is also shown as the equivalent USD per Euro.

Table S4.2 - 2024 annual-average exchange rates used for USD-equivalent orientation values

Currency pair	2024 annual average	Source
Canadian dollar	1.370 CAD/USD	U.S. Federal Reserve G.5A annual release [77]
Euro	0.9242 EUR/USD, equivalent to 1.082 USD/EUR	U.S. Federal Reserve G.5A annual release [77]

Currency pair	2024 annual average	Source
Japanese yen	151.5 JPY/USD	U.S. Federal Reserve G.5A annual release [77]
Chinese yuan	7.196 CNY/USD	U.S. Federal Reserve G.5A annual release [77]
Thai baht	35.28 THB/USD	U.S. Federal Reserve G.5A annual release [77]
Taiwan dollar	32.11 TWD/USD	U.S. Federal Reserve G.5A annual release [77]

Why USD-equivalent values are orientation values only

Exchange-rate conversion does not adjust for differences in healthcare purchasing power or opportunity cost across health systems. Two USD-equivalent ICERs from different jurisdictions can therefore imply very different real resource use.

Willingness-to-pay thresholds are jurisdiction-specific. The same USD-equivalent ICER may be considered acceptable in one setting and unacceptable in another because local thresholds, budget constraints, payer perspectives, and reimbursement processes differ.

The included studies use different perspectives, model structures, time horizons, clinical inputs, drug prices, utility values, and assumptions about subsequent therapy. USD-equivalent values therefore support readability but do not create a common ranking metric.

Numerical formatting convention

English-language numerical formatting is used throughout this supplement: comma (,) for thousands separators and period (.) for decimal points. Currency symbols are placed immediately before the value or currency abbreviation. Values that previously appeared in European-style formatting with a dot as a thousands separator have been reformatted to avoid ambiguity.

S4.2 Per-study conversion table

The original per-study conversion table has been split into two narrower tables to improve readability in portrait format. Table S4.3a reports source values and inflation inputs. Table S4.3b reports exchange rates, USD-equivalent orientation values, and interpretive notes. These values are not intended for cross-country ranking.

Table S4.3a - Per-study conversion table: source values and inflation inputs

#	Study	Original currency	Original price year	Headline value (original)	CPI ratio to 2024
1	Chamberlain 2021	USD	2019	$7,743 per patient-month total cost	1.2270, US 2019 to 2024
2	Chen R 2022	CNY	2021 or as reported	Cost difference for XELOX vs GEMOX reported in source study	1.0045, China 2022 to 2024 where applicable
3	Chen KA 2024	TWD (NT$)	2023	NT$6,268,528/QALY for ivosidenib; NT$5,670,555/QALY for 5-FU/LV comparator analysis	1.0220, Taiwan 2023 to 2024
4	Choi 2024	CAD	2021	Median CAD8,507/admission, IQR CAD5,416 to CAD16,152	1.1359, Canada 2021 to 2024
5	Chueh 2023	TWD (NT$)	2022	Pemigatinib NT$17,820 per 13.5 mg; ICER NT$3,411,098/QALY	1.0474, Taiwan 2022 to 2024
6	Darba and Marsa 2021	EUR	2018	EUR 9,417 per patient	1.1934, Spain 2018 to 2024
7	Jiang 2025	USD	2024	US$810,184/QALY, US arm; US$360,933/QALY, China arm	1.0000
8	Kashiwa and Maeda 2024	JPY	2024	JPY 3.78M/QALY GCS; JPY 86.06M/QALY DGC; JPY 28.98M/QALY PGC	1.0000
9	Laopachee 2023	THB	2022	approx. THB 152,985/QALY	1.0163, Thailand 2022 to 2024
10	Lundgren 2020	EUR	2018	EUR 76,508/LY, selective vs routine pathology	1.2642, Sweden 2018 to 2024
11	Luo X 2024	USD	2024	US$556,689/QALY, China arm; US$1,109,463/QALY, US arm	1.0000
12	Olthof 2018	EUR	2017	EUR 160,000/year aggregate centre-level cost	1.2813, Netherlands 2017 to 2024
13	Parasuraman 2023	USD	2021	Direct costs $10,300 to $11,200/month; indirect costs $622 to $690/month	1.1577, US 2021 to 2024
14	Roth and Carlson 2012	USD	2010	ICER approx. US$59,480/QALY	1.4386, US 2010 to 2024
15	Tsukiyama 2017	JPY	2015	ICER approx. JPY 13.7M/QALY	1.1044, Japan 2015 to 2024
16	Wadhwa 2017	USD	1997 to 2012, source reports inflation-adjusted trend	Mean charges increased from $36,460 to $77,753	1.3663, US 2012 to 2024 for endpoint
17	Wang 2024	USD	2023	1L $19,589 PPPM; 2L $22,617 PPPM; 3L $33,534 PPPM	1.0295, US 2023 to 2024
18	Ye 2023	USD	2023	US$381,864/QALY, US arm; US$367,609/QALY, China arm	1.0295, US 2023 to 2024
19	Zhao 2023	CNY	2022	ICER with charity approx. CNY 1.06M/QALY; without charity approx. CNY 4.7M/QALY	1.0045, China 2022 to 2024
20	Zheng 2023	CNY / USD as reported	2022	ICER reported by source study approx. US$564,895/QALY	1.0045, China 2022 to 2024

Table S4.3b - Per-study conversion table: exchange rates, USD-equivalent orientation values, and notes

#	Study	Exchange rate	USD 2024 equivalent (orientation only)	Notes
1	Chamberlain 2021	n/a, USD	$9,501 per patient-month	Total cost reported in 2019 USD; 2024 equivalent calculated using U.S. CPI.

#	Study	Exchange rate	USD 2024 equivalent (orientation only)	Notes
2	Chen R 2022	7.196 CNY/USD	As reported by source study	Source study reports headline costs and cost-effectiveness results; conversion is used only for orientation where values are discussed narratively.
3	Chen KA 2024	32.11 TWD/USD	approx. US$199,500/QALY; approx. US$180,500/QALY	ICERs exceed the Taiwanese WTP threshold used in the study; source study reports price-reduction scenarios.
4	Choi 2024	1.370 CAD/USD	approx. US$7,053 per admission	Orientation value only; hospital-cost context and Canadian setting should be preserved in interpretation.
5	Chueh 2023	32.11 TWD/USD	approx. US$111,300/QALY	The source study reports that substantial price reduction improves cost-effectiveness probability.
6	Darba and Marsa 2021	0.9242 EUR/USD	approx. US$12,160 per patient	Inflated to EUR 11,239 in 2024 and converted using the 2024 EUR/USD orientation rate.
7	Jiang 2025	n/a, USD	As reported by source study	Study reports both ICERs in 2024 USD.
8	Kashiwa and Maeda 2024	151.5 JPY/USD	approx. US$24,950; approx. US$568,000; approx. US$191,300	GCS below the Japanese threshold used in the study; immunotherapy-containing regimens above threshold.
9	Laopachee 2023	35.28 THB/USD	approx. US$4,407/QALY	Orientation value only; key interpretation is relative to the Thai WTP threshold used by the authors.
10	Lundgren 2020	0.9242 EUR/USD	approx. US$104,650/LY	Study reports in EUR despite Swedish setting; value is pathway-adjacent, not treatment-level BTC economics.
11	Luo X 2024	n/a, USD	As reported by source study	Reported in 2024 USD; ICERs are above relevant jurisdictional thresholds.
12	Olthof 2018	0.9242 EUR/USD	approx. US$221,800/year	Centre-level descriptive cost; orientation value only.
13	Parasuraman 2023	n/a, USD	Direct approx. US$11,924 to US$12,966/month; indirect approx. US$720 to US$799/month	Productivity-loss estimates depend on source-study valuation methods.
14	Roth and Carlson 2012	n/a, USD	approx. US$85,566/QALY	Historical price year is important when comparing with newer studies.
15	Tsukiyama 2017	151.5 JPY/USD	approx. US$99,900/QALY	Interpretation should remain tied to the Japanese threshold and payer context used in the study.
16	Wadhwa 2017	n/a, USD	approx. US$106,200 for the 2012 endpoint	Trend interpretation only; not used for cross-study ranking.
17	Wang 2024	n/a, USD	1L approx. US$20,167; 2L approx. US$23,284; 3L approx. US$34,523	Per-patient-per-month costs stratified by treatment line.
18	Ye 2023	n/a, USD	approx. US$393,100/QALY; approx. US$378,500/QALY	Source study reports both ICERs in USD; payer context differs by arm.
19	Zhao 2023	7.196 CNY/USD	approx. US$148,000/QALY; approx. US$656,000/QALY	Both ICERs exceed the Chinese WTP threshold used in the study; price reduction is a major driver.
20	Zheng 2023	7.196 CNY/USD where applicable	approx. US$567,400/QALY	Above the Chinese WTP threshold used in the study.

Worked example of the conversion convention

Using Choi 2024 as an example: the original value is CAD8,507 per admission in 2021 prices. Inflating to 2024 using the Canadian CPI ratio gives CAD8,507 x 1.1359 approx. CAD9,663. Converting at the 2024 annual-average exchange rate of 1.370 CAD per USD gives CAD9,663 / 1.370 approx. US$7,053.

This arithmetic is intentionally simple. The purpose of the supplement is to provide an audit trail for the orientation values rather than to re-estimate the source studies. Readers who prefer a different reference year, inflation index, or purchasing-power-parity conversion can apply the same approach with alternative assumptions.

S4.3 Relationship with Table 1

Table 1 in the main manuscript reports monetary values primarily in the original currency and price context used by each source study. Where USD equivalents are shown, they are orientation values only and should be interpreted together with the original currency, price year, payer perspective, comparator, and jurisdiction-specific willingness-to-pay threshold. The conversion assumptions used for retained USD-equivalent values are shown in Tables S4.3a and S4.3b.

The table and supplement use English-language numerical formatting. For example, values such as NT$3,411,098 and US$112,000 are treated as thousands-separated values, not decimal values. This convention is used to avoid ambiguity across source studies and reviewer comments.

S4.4 Relationship with Figure 2

Figure 2 uses USD-equivalent orientation values only where these are documented in Tables S4.3a and S4.3b or reported directly by the source study. No universal willingness-to-pay threshold is applied because thresholds differ across jurisdictions. Readers should interpret the plotted values alongside the original study currency, payer perspective, comparator, price year, and jurisdiction-specific threshold. The figure is intended as a visual summary of the direction and magnitude of published ICERs, not as a formal cross-country ranking of value.

S4.5 Limitations of this supplement

This supplement provides an audit trail for currency presentation but does not perform a re-analysis of any included study. It does not standardize ICERs in a way that supports cross-jurisdictional ranking.

General CPI indices were used for transparency and cross-country comparability, but healthcare-specific inflation may differ from general inflation. Exchange-rate conversion also does not account for purchasing-power parity, differences in health-system opportunity costs, confidential discounts, procurement arrangements, or local reimbursement rules.

For these reasons, original-currency and jurisdiction-specific interpretations remain primary. USD-equivalent values are secondary orientation values only.

References for Supplementary Material S4

Studies cited in this supplement use the same reference numbers as the main manuscript reference list. Full bibliographic details for the included studies are provided in the manuscript reference list. The supplementary data-source references are listed below in the numbering style used in the main manuscript.

40. Page, M.J.; McKenzie, J.E.; Bossuyt, P.M.; Boutron, I.; Hoffmann, T.C.; Mulrow, C.D.; Shamseer, L.; Tetzlaff, J.M.; Akl, E.A.; Brennan, S.E.; et al. The PRISMA 2020 statement: An updated guideline for reporting systematic reviews. BMJ 2021, 372, n71. https://doi.org/10.1136/bmj.n71.

41. Husereau, D.; Drummond, M.; Augustovski, F.; de Bekker-Grob, E.; Briggs, A.H.; Carswell, C.; Caulley, L.; Chaiyakunapruk, N.; Greenberg, D.; Loder, E.; et al. Consolidated Health Economic Evaluation Reporting Standards 2022 (CHEERS 2022) Statement: Updated Reporting Guidance for Health Economic Evaluations. BMJ 2022, 376, e067975. https://doi.org/10.1136/bmj-2021-067975.

76. World Bank. World Development Indicators: Consumer Price Index (FP.CPI.TOTL). Available online: https://data.worldbank.org/indicator/FP.CPI.TOTL (accessed on 22 June 2026).

77. Board of Governors of the Federal Reserve System. Foreign Exchange Rates: G.5A Annual Release. Available online: https://www.federalreserve.gov/releases/g5a/current/ (accessed on 22 June 2026).

78. Directorate-General of Budget, Accounting and Statistics, Executive Yuan, Taiwan. Consumer Price Index. Available online: https://eng.dgbas.gov.tw/ (accessed on 22 June 2026).

79. Bank of Thailand; Ministry of Commerce, Thailand. Headline Consumer Price Index Annual Change. Available online: https://www.bot.or.th/ (accessed on 22 June 2026).
